# Nuclear position dictates DNA repair pathway choice

**DOI:** 10.1101/gad.248369.114

**Published:** 2014-11-15

**Authors:** Charlène Lemaître, Anastazja Grabarz, Katerina Tsouroula, Leonid Andronov, Audrey Furst, Tibor Pankotai, Vincent Heyer, Mélanie Rogier, Kathleen M. Attwood, Pascal Kessler, Graham Dellaire, Bruno Klaholz, Bernardo Reina-San-Martin, Evi Soutoglou

**Affiliations:** 1 Institut de Génétique et de Biologie Moléculaire et Cellulaire (IGBMC), 67404 Illkirch CEDEX, France;; 2 U964, Institut National de la Santé et de la Recherche Médicale (INSERM), 67404 Illkirch CEDEX, France;; 3 UMR7104, Centre National de Recherche Scientifique (CNRS), 67404 Illkirch CEDEX, France;; 4 Université de Strasbourg (UDS), 67404 Illkirch CEDEX, France;; 5 Department of Pathology,; 6 Department of Biochemistry and Molecular Biology, Dalhousie University, Halifax, Nova Scotia B3H 4R2, Canada

**Keywords:** alternative end-joining, DNA repair, nuclear lamina, nuclear organization

## Abstract

Faithful DNA repair is essential to avoid chromosomal rearrangements and promote genome integrity. Nuclear organization has emerged as a key parameter in the formation of chromosomal translocations, yet little is known as to whether DNA repair can efficiently occur throughout the nucleus and whether it is affected by the location of the lesion. Here, we induce DNA double-strand breaks (DSBs) at different nuclear compartments and follow their fate. We demonstrate that DSBs induced at the nuclear membrane (but not at nuclear pores or nuclear interior) fail to rapidly activate the DNA damage response (DDR) and repair by homologous recombination (HR). Real-time and superresolution imaging reveal that DNA DSBs within lamina-associated domains do not migrate to more permissive environments for HR, like the nuclear pores or the nuclear interior, but instead are repaired in situ by alternative end-joining. Our results are consistent with a model in which nuclear position dictates the choice of DNA repair pathway, thus revealing a new level of regulation in DSB repair controlled by spatial organization of DNA within the nucleus.

## Abstract (precis)

Faithful DNA repair is essential to avoid chromosomal rearrangements and promote genome integrity. Lemaitre et al. demonstrate that double-strand breaks (DSBs) induced at the nuclear membrane fail to rapidly activate the DNA damage response and repair by homologous recombination (HR). DNA DSBs within lamina-associated domains do not migrate to more permissive environments for HR, like the nuclear pores or the nuclear interior, but instead are repaired in situ by alternative end-joining.

Cells continuously experience stress and damage from exogenous sources, such as UV light or irradiation, and endogenous sources, such as oxidative by-products of cellular metabolism (Jackson and Bartek 2009). To avoid subsequent genomic instability, several pathways evolved to detect DNA damage, signal its presence, and mediate its repair (Misteli and Soutoglou 2009). The two main pathways for double-strand break (DSB) repair are homologous recombination (HR) and nonhomologous end-joining (NHEJ) (Chapman et al. 2012).

DNA repair occurs in the highly compartmentalized nucleus, and emerging evidence suggests an important role of nuclear organization in the maintenance of genome integrity (Misteli and Soutoglou 2009). Observations in yeast suggest that distinct, dedicated DNA repair centers exist as preferential sites of repair (Lisby et al. 2003). Further evidence for spatially restricted repair in yeast comes from the observation that persistent DSBs migrate from their internal nuclear positions to the nuclear periphery, where they associate with nuclear pores (Therizols et al. 2006; Nagai et al. 2008; Oza et al. 2009). In mammalian cells, multiple DSBs on several chromosomes are repaired individually and do not meet on shared repair centers or move toward the nuclear periphery (Soutoglou et al. 2007). In line with these observations, spatial proximity of DSBs in the nucleus is a key parameter that affects the frequency of formation of chromosomal translocations in mammals (Roukos et al. 2013; Roukos and Misteli 2014). Therefore, in mammals, although nuclear organization has emerged as a key parameter in the formation of chromosomal translocations (for review, see Roukos and Misteli 2014), very little is known about how nuclear compartmentalization contributes to genome stability and whether DNA repair occurs throughout the nucleus with the same robustness and accuracy.

Here, we used an inducible system to create temporally and spatially defined DSBs in chromatin within different nuclear compartments and followed their fate. We show that the presence of heterochromatin at the nuclear lamina delays DNA damage response (DDR) and impairs HR. We further used live-cell imaging and superresolution microscopy to probe the spatial dynamics of these DSBs. We show that, contrary to what was observed in yeast, DNA DSBs within lamina-associated domains (LADs) do not migrate to more permissive environments for HR, like the nuclear pores or the nuclear interior. Instead, they are repaired in situ by NHEJ or alternative end-joining (A-EJ). Our data reveal a new level of regulation in DSB repair pathway choice controlled by spatial organization of DNA in the nucleus.

## Results

To investigate the impact of nuclear compartmentalization on DNA repair, we induced DSBs in chromatin associated with the inner nuclear membrane and then tested the consequences of nuclear position in DDR kinetics and DNA repair efficiency. We generated I-U2OS19 cells that contain a stably integrated I-SceI restriction site flanked by 256 repeats of the lac operator DNA sequences (lacO) (Supplemental Fig. S1A). This cell line was also engineered to express the I-SceI endonuclease under the control of a doxycycline (Dox)-inducible promoter (pTRE-tight), allowing us to temporally control the induction of a DSB at the lacO/I-SceI locus (Supplemental Fig. S1A). Stable expression of the GFP lac repressor (lacI) enables the visualization of the lacO/I-SceI locus in the nucleus. We induced specific tethering of the lacO locus at the inner nuclear membrane by the expression of an Emerin C-terminal deletion (ΔEMD), which localizes at the nuclear lamina, fused to GFP-lacI (GFP-lacI-ΔEMD) (Supplemental Fig. S1A) as described in Reddy et al. (2008).

Consistent with previous results (Reddy et al. 2008), ΔEMD is sufficient to target the GFP-lacI-ΔEMD fusion protein to the nuclear membrane and relocate the lacO/I-SceI-containing chromosome at the nuclear lamina after one mitotic cycle (Supplemental Fig. S1B,C). Indeed, in cells expressing GFP-lacI-ΔEMD, we observed 70% of colocalization of the lacO array with laminB by immuno-FISH in the absence or presence of I-SceI, whereas in cells expressing GFP-lacI, this colocalization is as low as 10% (Supplemental Fig. S1B,C).

To determine whether tethering of the lacO/I-SceI locus to the nuclear lamina has an effect on the accessibility of the I-SceI endonuclease, we performed ligation-mediated PCR (LM-PCR) in cells expressing GFP-lacI or GFP-lacI-ΔEMD. We found that the cutting efficiency is equivalent in both environments (Supplemental Fig. S1D), demonstrating that the I-SceI endonuclease is able to recognize its target sequence and cleave its substrate regardless of its nuclear localization.

DSBs activate the DDR, which allows recognition of breaks and the activation of checkpoints. Consequently, cell cycle progression is paused, which allows time for the cell to repair the lesions before dividing (Misteli and Soutoglou 2009). DDR involves a megabase-wide spreading of a phosphorylated form of the histone variant H2AX (γ-H2AX) around them (Rogakou et al. 1998; Misteli and Soutoglou 2009).

**Figure 1.**
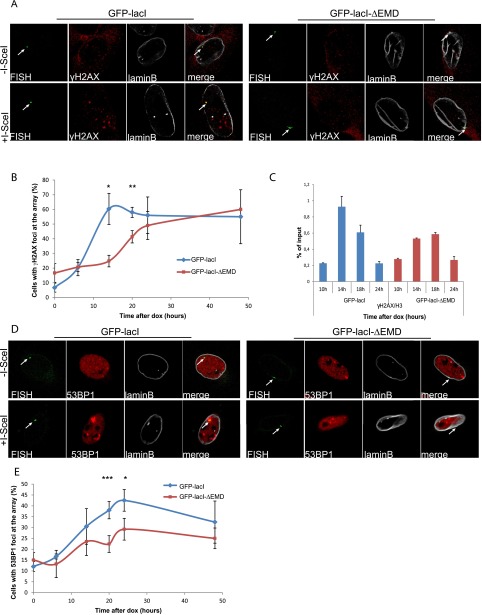
The DDR is delayed at the nuclear lamina. (*A*) Immuno-FISH single-Z confocal images of the lacO array (green), γ-H2AX (red), and laminB (gray) in I-U2OS19 cells expressing GFP-lacI or GFP-lacI-ΔEMD and treated or not with Dox for 14 h. (*B*) Time course of the percentage of colocalization of the lacO array with γ-H2AX. (*C*) γ-H2AX ChIP at the indicated time points after Dox addition in cells expressing GFP-lacI or GFP-lacI-ΔEMD. Values were normalized to input DNA and H3 ChIP and are representative of three independent experiments. (*D*) Immuno-FISH single-Z confocal images of the lacO array (green), 53BP1 (red), and laminB (gray) in I-U2OS19 cells expressing GFP-lacI or GFP-lacI-ΔEMD and treated or not with Dox for 20 h. (*E*) 53BP1 after Dox addition in I-U2OS19 cells expressing GFP-lacI or GFP-lacI-ΔEMD. Values represent mean ± SD of three independent experiments with *n* > 50 cells. For statistical analysis, a *t*-test was performed. (*) *P* < 0.05; (**) *P* < 0.01; (***) *P* < 0.001. In all figures, the arrow depicts the position of the lacO array.

To assess the impact of repositioning the lacO/I-SceI locus at the nuclear lamina compartment on DDR efficiency, we compared the kinetics of induction of γ-H2AX at the I-SceI break in cells expressing GFP-lacI or GFP-lacI-ΔEMD by immuno-FISH. Although repositioning of the lacO/I-SceI break at the nuclear lamina did not affect the maximal percentage of γ-H2AX, cells expressing GFP-lacI showed the highest percentage of γ-H2AX colocalization with the lacO/I-sceI locus 14 h after Dox addition, whereas GFP-lacI-ΔEMD cells only achieved the same level 24 h after Dox was added (Fig. 1A,B). This observation was further confirmed by chromatin immunoprecipitation (ChIP) experiments (Fig. 1C). We also investigated the recruitment of another DDR factor, 53BP1, which has been implicated in the choice of the DSB repair pathway (Bunting et al. 2010; Panier and Boulton 2014). Similarly to γ-H2AX, the recruitment of 53BP1 was also delayed and showed a maximal accumulation at 24 h after I-SceI expression in GFP-lacI-ΔEMD cells compared with 20 h in GFP-lacI cells (Fig. 1D,E). A similar difference was observed in a lacO/I-SceI system integrated in the I-Hela111 cell line (Supplemental Fig. S2A,B), suggesting that the effect is not tissue-specific but rather is a general mechanism. Taken together, these results reveal a general delay in DDR in lesions occurring in chromatin associated with the nuclear lamina and suggest that this compartment is a repressive microenvironment for DDR.

To rule out the possibility that this defect was due to the expression of the ΔEMD in the context of the GFP-lacI-ΔEMD fusion protein, we performed an immuno-FISH experiment in the presence of IPTG. Under these conditions, the GFP-lacI-ΔEMD fusion protein is expressed but does not bind to the lacO array, and the array is not relocalized at the nuclear lamina, which was confirmed by the markedly reduced colocalization of the array and laminB (Supplemental Fig. S3A–C). As shown in Supplemental Figure S3B and quantified in Supplemental Figure S3D, there was no difference in the degree of γ-H2AX at the I-SceI break in cells expressing either GFP-lacI or GFP-lacI-ΔEMD in the presence of IPTG and 14 h after Dox where there was the maximal difference in DDR between the two compartments (Fig. 1B), confirming that the decreased phosphorylation of H2AX is a consequence of a lesion induced at the nuclear lamina.

**Figure 2.**
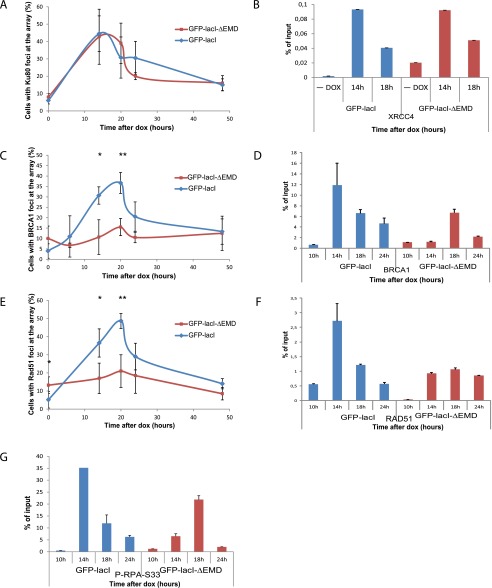
Recruitment of HR factors is impaired at the nuclear lamina. (*A*) Time course of the percentage of colocalization of the lacO array with Ku80 after Dox addition in I-U2OS19 cells expressing GFP-lacI or GFP-lacI-ΔEMD. Values represent mean ± SD of three independent experiments with *n* > 50 cells. ChIP for XRRC4 (*B*), BRCA1 (*D*), RAD51 (*F*), or P-RPAS33 (*G*) at the indicated times upon Dox addition in I-Hela111 cells (XRCC4) or I-U2OS19 cells expressing GFP-lacI or GFP-lacI-ΔEMD is shown. Values were normalized to input DNA and are representative of three independent experiments. The percentage of colocalization of the lacO array with BRCA1 (*C*) and Rad51 (*E*) at the indicated times after Dox addition in I-U2OS19 cells expressing GFP-lacI or GFP-lacI-ΔEMD is shown. Values represent mean ± SD of three independent experiments with *n* > 50 cells. For statistical analysis, a *t*-test was performed. (*) *P* < 0.05; (**) *P* < 0.01.

In light of the above observations, we investigated whether the delay in DDR at the I-SceI lesion at the nuclear membrane impacts on its repair. To evaluate the effect of the I-SceI break repositioning at the inner nuclear membrane on NHEJ, we compared the degree of colocalization of Ku80 (Britton et al. 2013) with the lacO/I-SceI array by immuno-FISH and the recruitment of XRCC4 by ChIP in cells expressing GFP-lacI and GFP-lacI-ΔEMD, two main proteins of the NHEJ pathway (Lieber 2010). We observed no difference in the recruitment of KU80 in I-U2OS19 (Fig. 2A; Supplemental Fig. S4A) and I-Hela111 (Supplemental Fig. S5A–D) cells or XRCC4 at I-Hela111 (Fig. 2B) at the I-SceI break induced at the nuclear lamina compared with the nuclear interior, suggesting that NHEJ can occur efficiently in both compartments. Interestingly, the recruitment of NHEJ factors was not delayed, which is indicative of an uncoupling of DDR and repair by NHEJ.

HR is mainly active during the S phase of the cell cycle and uses the homologous sister chromatid as a template for error-free repair (San Filippo et al. 2008). Contrary to what was observed for NHEJ proteins, the recruitment of HR factors such as BRCA1, Rad51 (Fig. 2C–F; Supplemental Figs. S4B,C, S5B,C,E,F), and Rad54 (Supplemental Fig. S6A) at the broken lacO residing at the inner nuclear membrane was markedly decreased. Interestingly, the phosphorylation of RPA was delayed and less robust but not entirely abolished, suggesting a semifunctional resection pathway (Fig. 2G) and a more dramatic effect specific to late HR factors. To verify that this difference was not due to an impaired cell cycle progression in the cells expressing GFP-lacI-ΔEMD, we compared the cell cycle profiles of the two cell lines by flow cytometry and observed no difference (Supplemental Fig. S6B). Our results suggest that the nuclear lamina is a repressive environment for HR.

In the mammalian nucleus, chromatin is organized into structural domains by association with distinct nuclear compartments (Parada and Misteli 2002; Bickmore 2013). To gain insight into the cause of the DDR delay and HR repression promoted by the nuclear lamina environment, we considered the possibility that the repressive chromatin structure associated with the nuclear lamina (Padeken and Heun 2014) is involved in this phenomenon (Goodarzi and Jeggo 2012; Lemaître and Soutoglou 2014).

**Figure 3.**
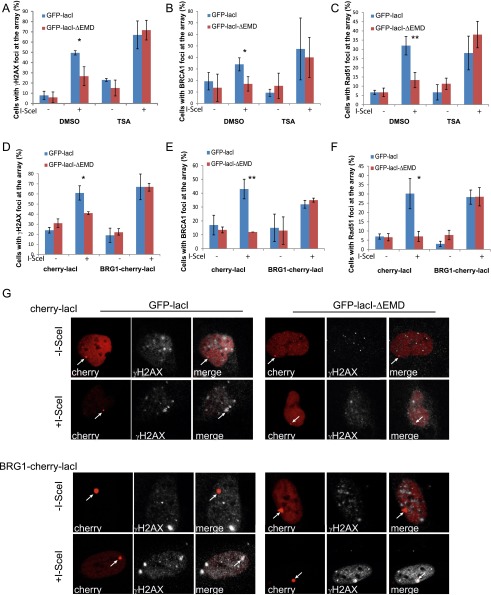
Chromatin decompaction restores DDR and the recruitment of HR factors at the nuclear lamina. Colocalization of the lacO array with γ-H2AX (*A*), BRCA1 (*B*), or RAD51 (*C*) in I-U2OS19 cells expressing GFP-lacI or GFP-lacI-ΔEMD and pretreated for 4 h with DMSO or TSA in the absence or presence of Dox for 14 h or 20 h is shown. The percentage of colocalization of the lacO array with γ-H2AX (*D*), BRCA1 (*E*), or RAD51 (*F*) in I-U2OS19 cells expressing GFP-lacI or GFP-lacI-ΔEMD and cherry-LacI or BRG1-cherry-lacI and treated or not with Dox for 14 h or 20 h is shown. (*G*) Immunofluorescence single-Z confocal images of γ-H2AX (gray) in I-U2OS19 cells expressing GFP-lacI or GFP-lacI-ΔEMD transfected with cherry-lacI or BRG1-cherry-lacI (red) and treated or not with Dox for 14 h. For statistical analysis, a *t*-test was performed. (*) *P* < 0.05; (**) *P* < 0.01.

To test this hypothesis, we treated cells with an inhibitor of histone deacetylases, trichostatin A (TSA). This treatment resulted in an increase in histone acetylation (Supplemental Fig. S7A) and loss of heterochromatin in the nucleus, including perinuclear heterochromatin, leading to a homogenous chromatin state, as visualized by electron microscopy (Supplemental Fig. S7B–D). TSA treatment did not perturb the repositioning of the lacO/I-SceI locus at the inner nuclear membrane (Supplemental Fig. S7E,F). Interestingly, TSA treatment rescued the defect in γ-H2AX and recruitment of BRCA1 and RAD51 observed after the lacO locus relocalization at the inner nuclear membrane, pointing to an inhibitory role of chromatin compaction in DDR and HR (Fig. 3A–C; Supplemental Figs. S8, S9A,B). Our results are in line with previous studies that showed that reduced gene expression around the nuclear periphery after repositioning of the lacO array depends on the activity of histone deacetylases (Finlan et al. 2008).

To further confirm that the perinuclear heterochromatin in contact with the nuclear membrane is responsible for delayed DDR and repressed HR, we induced decondensation of the lacO/I-SceI chromatin by direct tethering of the chromatin remodeler BRG1. To this end, we expressed cherry-lacI-BRG1 in cells expressing GFP-lacI or GFP-lacI-ΔEMD (Supplemental Fig. S10A). As shown in Supplemental Figure S10B and quantified in Supplemental Figure S10C, tethering of BRG1 at the lacO array resulted in local chromatin decondensation, as visualized by an increased size of the array.

Similar to what we observed after global chromatin decondensation, local chromatin opening by BRG1 rescued the defect in γ-H2AX and the recruitment of BRCA1 and RAD51 upon lacO repositioning at the lamina (Fig. 3D–G; Supplemental Fig. S11A,B). Altogether, these results strongly suggest that the decreased recruitment of HR factors at the nuclear lamina is due to the highly compacted state of the surrounding chromatin.

**Figure 4.**
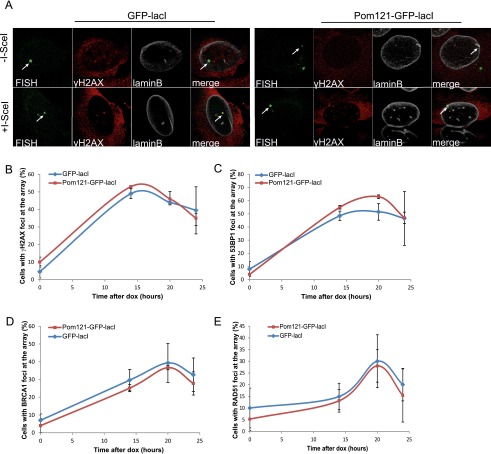
DDR and HR are not affected by tethering at the nuclear pores. (*A*) Immuno-FISH single-Z confocal images of the lacO array (green), γ-H2AX (red), and laminB (gray) in I-U2OS19 cells expressing GFP-lacI or Pom121-GFP-lacI and treated or not with Dox for 14 h. Time course of the percentage of colocalization of the lacO array with γ-H2AX (*B*), 53BP1 (*C*), BRCA1 (*D*), or RAD51 (*E*) in I-U2OS19 cells expressing GFP-lacI or Pom121-GFP-lacI cells after Dox addition is shown. Values represent mean ± SD of three independent experiments with *n* > 50 cells.

To further examine whether the localization of a DSB within a nuclear compartment in relation to the state of the chromatin that surrounds the compartment can influence the DNA repair pathway choice, we assessed DSB repair at the nuclear pores, which are subcompartments of the nuclear periphery that represent a permissive environment for gene expression and other DNA-dependent nuclear transactions (Taddei et al. 2006; Ptak et al. 2014). To position the lacO/I-SceI locus at the nuclear pore compartment, we expressed GFP-lacI fused to the nucleoporin Pom121 (Supplemental Fig. S12A). We found that repositioning of the lacO array to the nuclear pores did not affect DDR, as visualized by H2AX phosphorylation and 53BP1 recruitment (Fig. 4A–C; Supplemental Fig. S12B). Furthermore, the recruitment of HR factors was similar in cells expressing GFP-lacI and GFP-lacI-Pom121 (Fig. 4D,E; Supplemental Fig. S12C,D). These observations suggest that in contrast to the nuclear lamina, nuclear pores represent a permissive microenvironment for DDR and DSB repair by HR. Therefore, although the nuclear lamina and nuclear pores are in very close proximity in the nuclear periphery, the difference in chromatin compaction associated with the two compartments regulates the choice of the repair pathway that will be prevalent in lesions occurring in each compartment.

It was previously shown that breaks inflicted at pericentric heterochromatin in *Drosophila* migrate at the periphery of the heterochromatin domain for HR repair in order to avoid recombination between repetitive sequences (Chiolo et al. 2011). Given that tethering of the lacO/I-SceI locus at the nuclear membrane using the GFP-lacI-ΔEMD might limit its potential mobility toward activating environments for DDR and repair, such as the nucleoplasm or the nuclear pores, we asked whether the lacO/I-SceI locus acquires mobility after break induction in the presence of IPTG when the lacI is not bound to the lacO array and cannot constrain its movement (Supplemental Fig. S13A). Surprisingly, we did not detect any migration of I-SceI breaks away from the compartment (Supplemental Fig. S13B).

**Figure 5.**
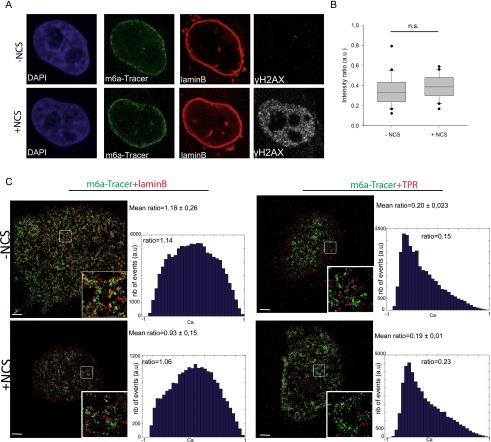
DSBs at the nuclear lamina are positionally stable. (*A*) Immunofluorescence of HT1080 cells expressing Dam-LaminB1 and m6A-Tracer 2 h after treatment (or not) with 50 ng/mL neocarzinostatin (NCS) for 15 min. (*B*) Box plot of GFP intensity ratios of the signal in the nucleoplasm versus the signal at the nuclear envelope in a HT1080-derived clonal cell line expressing a Dam-LaminB1 and the m6A-Tracer. The number of cells analyzed per condition was 20. For statistical analysis, χ^2^ tests were performed. (n.s.) Nonsignificant. (*C*) dSTORM microscopy images of LADs (green) and laminB (*left* panel; red) or TPR (*right* panel; red) in the absence (*top* panel) or presence (*bottom* panel) of DNA damage (100 ng/mL NCS for 15 min and released for 2 h) in HT1080 cells expressing Dam-LaminB1 and m6A-Tracer. Images were taken from the bottom of the cells to allow better resolution of nuclear pores. Corresponding colocalization and the ratio of positive over negative colocalization events are displayed at the *right*. The mean ratios for all nuclei analyzed (*n* ≥ 8) are displayed *above*.

To further investigate whether breaks occurring at the lamina migrate away from the lamina compartment toward the adjacent pores or the interior of the nucleus, we used an experimental system previously developed to visualize chromatin domains associated with laminB in single cells (Kind et al. 2013). This system uses DNA adenine methylation as a tag to visualize and track LADs using a truncated version of the DpnI enzyme fused to GFP (m6a-Tracer), which recognizes methylated LADs in cells expressing LaminB-Dam (Kind et al. 2013). To probe the behavior of LADs in the presence of DNA damage, we followed the m6a-Tracer localization using live-cell imaging (Supplemental Fig. S13C) or confocal (Fig. 5A,B) or superresolution (Fig. 5C) microscopy. The infliction of DNA damage in the LADs was verified by γ-H2AX (Fig. 5A; Supplemental Fig. S13D). Interestingly, the partition of the LADs between the nuclear membrane and the nucleoplasm did not notably change before and after global DNA damage (Fig. 5A–C; Supplemental Fig. S13C), suggesting that DNA lesions do not lead to massive rearrangements of LADs within the nucleus.

In yeast, persistent DSBs migrate from their internal nuclear positions to the nuclear periphery, where they associate with nuclear pores (Therizols et al. 2006; Nagai et al. 2008; Oza et al. 2009). To more precisely assess the spatial proximity of LADs with laminB and nucleoporin of the nuclear basket TPR before and after DNA damage, we used two-color dSTORM superresolution microscopy (Folling et al. 2008). As expected, we observed juxtaposition and a certain degree of colocalization of LADs with LaminB but not with TPR (Fig. 5A). Interestingly, DNA damage did not induce changes in the proximity of LADs toward both compartments, which further pointed to the positional stability of LADs upon DNA damage (Fig. 5A). Taken together, these results suggest that contrary to what has been shown in yeast, breaks occurring on chromosomes that associate with the nuclear membrane do not travel and seek an environment permissive to HR repair, such as the nuclear pores.

**Figure 6.**
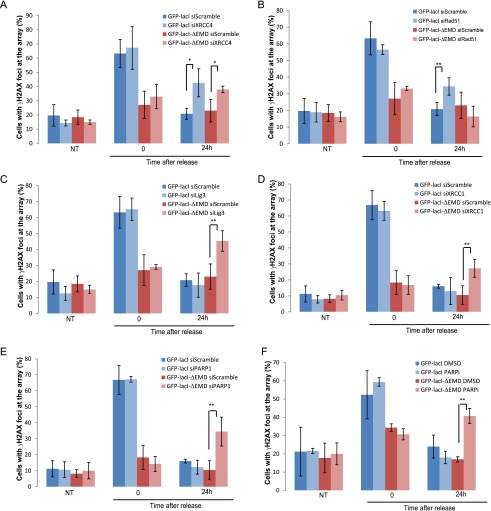
DSBs at the nuclear lamina are repaired by NHEJ or A-EJ. The percentage of colocalization of the lacO array with γ-H2AX in untreated cells (NT) or after 14 h of Dox (time point 0) and subsequent release for 24 h in I-U2OS19 cells expressing GFP-lacI or GFP-lacI-ΔEMD and transfected with XRCC4 (*A*), RAD51 (*B*), ligase 3 (*C*), XRCC1 (*D*), or PARP1-specific siRNAs (*E*) is shown. (*F*) The percentage of colocalization of the lacO array with γ-H2AX upon Dox treatment or release in I-U2OS19 cells expressing GFP-lacI or GFP-lacI-ΔEMD and treated with DMSO or a PARP inhibitor (PARPi, during the entire course of the experiment) is shown. Values represent mean ± SD of three independent experiments with *n* > 50 cells. For statistical analysis, a *t*-test was performed. (*) *P* < 0.05; (**) *P* < 0.01.

To further investigate the contribution of NHEJ and HR in repairing the I-SceI breaks at the lamina or the nuclear interior, we assessed the degree of persistent breaks in GFP-lacI or GFP-lacI-ΔEMD cells depleted of XRCC4 and RAD51 (knockdown efficiencies verified in Supplemental Fig. S14A). Interestingly, in control cells, breaks were efficiently repaired in both nuclear compartments, which was exemplified by the decrease in γ-H2AX signal at the lacO array 24 h after break induction by a short pulse of Dox (Fig. 6A–E). Although depletion of XRCC4 led to persistent damage in both compartments (Fig. 6A), depletion of RAD51 did not affect the repair of breaks at the lamina (Fig. 6B). These results suggest that lesions at LADs do not depend on HR for their repair.

To test whether repositioning of the lacO/I-SceI break at the nuclear membrane affects the kinetics of repair, we performed LM-PCR in GFP-lacI and GFP-lacI-ΔEMD cells after a short pulse of Dox followed by release for 36 h. We found that breaks at both nuclear locations were efficiently repaired based on the marked decrease in PCR signal (Supplemental Fig. S14B). These results strongly suggest that efficient DNA repair takes place at the lamina-associated I-SceI breaks even in the absence of functional HR.

Since resection is not abolished at lacO/I-SceI breaks when associated with the nuclear lamina, we sought to determine the fate of the lesions whereby resection has occurred but complete DNA repair by HR cannot occur. To answer this question, we assessed the contribution of the A-EJ pathway in the repair of breaks at the periphery. To this end, we quantified persistent γ-H2AX at the lacO/I-SceI locus 24 h after break induction in GFP-lacI and GFP-lacI-ΔEMD cells where ligase 3, XRCC1, or PARP1 had been depleted (knockdown efficiencies verified in Supplemental Fig. S14A,C) or PARP was inhibited. Interestingly, inhibition of the A-EJ pathway resulted in a repair delay for only breaks that were associated with the nuclear membrane (Fig. 6C–F; Supplemental Fig. S14D). These findings indicate that NHEJ and A-EJ, but not HR, are the most prevalent pathways of DNA repair for lesions occurring at nuclear membrane-associated chromatin and reveal for the first time that A-EJ takes place as a main pathway and not as a backup pathway activated solely in instances where there is a DNA repair factor deficiency (Frit et al. 2014).

Taken together, we showed that breaks occurring in chromatin that surrounds the nuclear membrane do not migrate to other regions of the nucleus, not even to other domains within the nuclear periphery, but rather are repaired within the lamina, where the break occurred by NHEJ and A-EJ.

## Discussion

To preserve genomic integrity, different DNA repair pathways have evolved, and multiple layers of regulation like the cell cycle, specific proteins, or chromatin structure exist to ensure the tight balance between these pathways (Kass and Jasin 2010). Here, we propose another layer of regulation of DNA repair pathway choice imposed by nuclear compartmentalization. We show that the nuclear lamina restricts HR and allows NHEJ and A-EJ. These observations are in agreement with data in yeast showing that distinct nuclear compartments of the nuclear periphery like the nuclear pore or the inner nuclear membrane favor different repair outcomes (Nagai et al. 2008; Khadaroo et al. 2009; Oza et al. 2009; Horigome et al. 2014). Similar to what we observed, it was shown that binding of DSBs to Nup84 in yeast facilitates recombination through SUMO protease Ulp1 and the SUMO-dependent ubiquitin ligase Slx5/Slx8 (Nagai et al. 2008) using BIR and microhomology-mediated recombination. On the contrary, binding to the inner nuclear membrane protein Mps3 has two different outcomes: In the case of telomere tethering, it inhibits recombination by sequestering the DSBs from nonspecific interactions with chromatin (Oza et al. 2009; Schober et al. 2009), while in the case of persistent DSBs, it triggers repair by the classical HR pathway (Horigome et al. 2014).

We also found that the chromatin structure at the inner nuclear lamina is mainly responsible for inhibiting HR. This is in keeping with recent studies, which found that HR is activated at DSBs located within actively transcribed genes that reside in euchromatin (Aymard et al. 2014; Pfister et al. 2014). Given that the lacO locus is promoterless and not transcribed, our results indicate that HR is not regulated solely by the transcriptional status. Instead, the exact nature of the chromatin environment and chromatin accessibility appear to be major determinants of HR regulation (Jha and Strahl 2014; Pai et al. 2014). Indeed, other studies have shown that HR is a main pathway in repairing breaks within heterochromatin (Beucher et al. 2009; Geuting et al. 2013; Kakarougkas et al. 2013). However, our data point to the fact that not all heterochromatin domains within the nucleus behave in the same manner and that the specific type of heterochromatin at the nuclear lamina has distinct functions.

In most of the above studies, chromatin structure and histone modifications affect the very first step of the HR pathway that is DNA end resection. Aymard et al. (2014) show that H3K36me3 is essential for the recruitment of CtIP through LEDGF. On the other hand, H3K36me3 in yeast induces chromatin compaction and inhibits resection, as visualized by increased RPA foci when the methyltransferase responsible for this modification is absent (Pai et al. 2014). Here we observed that phosphorylation of RPA at S33 is delayed and not mounted properly at lesions occurring in chromatin associated with the inner nuclear membrane. We also show that BRCA1 recruitment is dramatically affected. Since BRCA1 is acting with CtIP to activate long-term resection (Chen et al. 2008), it is possible that DNA ends are not appropriately resected to create a proper template for recombination, and the short resection channels lesions to A-EJ as was proposed earlier (Zhang and Jasin 2011; Deng et al. 2014). The fact that resection at the lamina is not as dramatically affected as late steps of HR might also suggest that nuclear position dictates the DNA repair pathway choice by regulating only the recruitment of late HR proteins to DSBs.

The use of A-EJ, which is considered a highly mutagenic pathway, instead of the error-free HR pathway might seem dangerous for the maintenance of genomic stability. However, LADs are relatively gene-poor, have a repressive chromatin signature, and are demarcated by repetitive and AT-rich sequences (Meuleman et al. 2013). The inhibition of HR may represent a means to avoid genomic instability provoked by recombination between repetitive sequences, which is a mechanism that has been proposed for the repair of DSBs that form in heterochromatic regions in *Drosophila* (Chiolo et al. 2011). Moreover, activation of A-EJ that is an error-prone pathway might have less impact given that most of the genes that reside in LADs are not transcribed (Meuleman et al. 2013).

In *Drosophila*, breaks induced in the heterochromatic domain rapidly relocate outside of the domain, where HR is completed (Chiolo et al. 2011). A similar DSB relocation was observed in mouse cells upon break induction by linear ion tracks in chromocenters (Jakob et al. 2011). On the contrary, we show that breaks occurring in chromatin associated with the inner nuclear lamina are positionally stable, suggesting that different heterochromatic compartments use different strategies to avoid recombination. One of the possible hypotheses to explain such a difference is a different chromatin composition or a difference in the regulation of chromatin mobility. Indeed, in yeast, DSBs were shown to have increased mobility (Dion and Gasser 2013). This mobility is facilitated by chromatin decompaction via chromatin remodelers (Neumann et al. 2012) and HR factors (Dion et al. 2012) and in turn allows the homology search step of HR (Mine-Hattab and Rothstein 2012). In mammalian cells, however, DSB mobility is limited and actively restricted by the NHEJ complex Ku70/Ku80 (Soutoglou et al. 2007; Roukos et al. 2013). In *Drosophila* cells, the relocation of DSBs outside of the heterochromatic domain is accompanied by decondensation of the domain (Chiolo et al. 2011), suggesting a mechanism similar to the one responsible for DSB mobility in yeast. At the nuclear lamina, however, this mechanism does not seem to be active, suggesting that an additional mechanism could repress DSB movement at the nuclear lamina. This hypothesis is in accordance with the observation that chromatin mobility is decreased for genomic loci associated with the nuclear lamina or the nucleoli (Chubb et al. 2002). Furthermore, laminA has recently been identified as a factor inhibiting DSB movement in mammalian cells (Mahen et al. 2013), further pointing to an active inhibition of DSB mobility at the nuclear lamina.

Another difference between our results and the results obtained in the heterochromatic compartment of *Drosophila* cells is the activation of DDR. In *Drosophila* cells, the activation of DDR was faster in heterochromatin compared with euchromatin (Chiolo et al. 2011). On the contrary, our results show a slower DDR activation at the nuclear lamina compared with the nuclear interior. Given the implication of the early steps of DDR in the initiation of resection by the ATM and MRN complexes, and the fact that resection facilitates DSB movement in yeast, one can hypothesize that the delayed DDR at the nuclear lamina inhibits DSB mobility.

Overall, our findings indicate that spatial positioning of a DSB is a new parameter to consider in the study of DSB repair, which has significant implications for our understanding of how the organization of repair in the highly compartmentalized nucleus contributes to maintaining genome stability and avoiding tumorigenesis.

## Materials and methods

### Cell lines, infections, transfections

I-U2OS19 GFP-lacI and GFP-lacI-ΔEMD cells were generated by infecting the U2OS19ptight13 cell line (Lemaître et al. 2012) with GFP-lacI (Soutoglou and Misteli 2008) and GFP-lacI-ΔEMD (Reddy et al. 2008) plasmids and after FACS sorting. Briefly, BOSC cells were transfected using FuGENE6 (Promega) according to the manufacturer’s protocol with GFP-lacI or GFP-lacI-ΔEMD constructs and an amphotropic vector. Cell supernatants were harvested 48 h later and transferred to U2OS19ptight13 cells. Twenty-four hours after infection, cells were FACS-sorted for GFP-positive signal and cultured in the presence of 800 μg/mL G418 and 2 mM IPTG (inhibitor of the lacI/lacO interaction). Cells were plated in the absence of IPTG for 24 h prior to starting an experiment. To induce I-SceI expression, Dox was added to the cells at a concentration of 1 μg/mL. In Supplemental Figure S3, 2 mM IPTG was maintained during the whole experiment, and in Supplemental Figure S7, A and B, cells were plated in the absence of IPTG for 24 h and treated with Dox for 12 h. IPTG was then added for 2 h, while Dox was maintained until the end of the experiment.

Hela111 cells were obtained by transfection of lacO-I-SceI-hygro plasmid and subsequent clonal selection using 300 μg/mL hygromycin. I-HeLa111 cells were generated by transfection of Hela111 cells with pWHE320-HA-I-SceI and pWHE146-Tet activator plasmids and selection using 1 mg/mL G418. I-Hela111 GFP-lacI or GFP-lacI-ΔEMD cells were generated by infection of I-Hela111 cells with GFP-lacI and GFP-lacI-ΔEMD plasmids and FACS sorting for GFP-positive cells.

I-U2OS19 Pom121-GFP-lacI cells were obtained after infection of I-U2OS19 cells with Pom121-GFP-lacI and selection of GFP-positive cells using FACs sorting.

I-U2OS19 GFP-lacI and GFP-lacI-ΔEMD were transfected with cherry-lacI or BRG1-cherry-lacI by using FuGENE6 reagent according to the manufacturer’s protocol. The cells were first plated in the absence of IPTG for 24 h and then transfected and treated with Dox 4 h after transfection.

I-U2OS19 GFP-lacI and GFP-lacI-ΔEMD cells were transfected with siRNA scramble (OnTarget Plus nontargeting pool siRNA; Dharmacon, D-001810-10-20), XRCC4 (Dharmacon, M-004494-02), Rad51 (Dharmacon, L-003530-00) or Lig3 (Dharmacon, L-009227-00) using oligofectamine reagent (Invitrogen) according to the manufacturer’s protocol. Knockdown efficiency was analysed by Western blot or RT-qPCR. RNA was extracted using the RNeasy minikit (Qiagen) according to the manufacturer’s protocol. RT-qPCRs were then processed as in (Pankotai et al. 2012). Proteins were extracted in RIPA buffer and analyzed by Western blot.

### PARP inhibitor treatment

I-U2OS19 GFP-lacI and GFP-lacI-ΔEMD were plated in the absence of IPTG for 24 h and treated with PARPi (ABT-888, sc-202901A) at a 10 μM concentration or by DMSO.

### TSA treatment

Cells were plated in the absence of IPTG for 24 h and subsequently treated with TSA at 0.5 μM or DMSO for control for 4 h. Dox was added after 4 h of treatment for the indicated time, while DMSO or TSA was maintained during the whole experiment.

### Neocarzinostatin (NCS) treatment

Cells were plated in the presence of Shield for 20 h, treated for 15 min with 100 ng/mL NCS (N9162-100UG, Sigma), and fixed 2 h after treatment.

### Cell cycle analysis

Cells were fixed in 70% EtOH overnight at −20°C and stained with 25 μg/mL propidium iodide. The acquisition was performed on a FACSCalibur. Results were analysed using FlowJo software.

### LM-PCR

Cells were plated in the absence of IPTG for 24 h and subsequently treated with Dox for 14 h. DNA was then extracted with the DNeasy blood and tissue kit (Qiagen). Assymetric adaptator (S21, Phos-GCATCACTACGATGTAGGATG; and Lup, CATCCTACATCGTAGTGATGCTTAT) was annealed in TE for 5 min at 95°C and then allowed to reach room temperature slowly. One-hundred picomoles of assymetric adaptator was added to 1 μg of DNA extracted from cells. Ligation was performed using T4 DNA ligase overnight at 16°C. PCR was performed using Pfu enzyme (Agilent) with an annealing temperature of 58°C. The PCR primers used were LM-I-SceI (CATCCTACATCGTAGTGATGC) and lacR (TTAATTAATCAAACCTTCCTCT). The PCR product was then run on a 2% agarose gel.

### Immunofluorescence, immuno-FISH, and microscopy

Cells were cultured on coverslips and fixed in 4% paraformaldehyde for 10 min, permeabilized in 0.5% Triton for 10 min, blocked in 1% BSA for 30 min, and incubated with primary antibody for 1 h (see the antibodies table in the Supplemental Material) and secondary antibodies for 45 min. Coverslips were incubated with DAPI and mounted on slides in Prolong Gold (Molecular Probes).

For Rad51 and Ku80 immunofluorescence or immuno-FISH, cells were pre-extracted in CSK buffer (10 mM Hepes at pH 7, 100 mM NaCl, 300 mM sucrose, 3 mM MgCl_2_, 0.7% Triton X-100) containing 0.3 mg/mL RNase A prior to fixation (Britton et al. 2013).

For immuno-FISH, the same protocol was used, but after incubation with secondary antibodies, they were submitted to post-fixation in 4% formaldehyde for 20 min. Cells were washed for 5 min in 2× SSC and 45 min in 2× SSC with a increasing temperature from room temperature to 72°C. After one wash in 70% ethanol and two washes in absolute ethanol, coverslips were dried for 5 min at room temperature. They were subsequently incubated with 0.1 N NaOH for 10 min and washed in 2× SSC for 5 min. Coverslips were washed again in 70% ethanol and twice with absolute ethanol. After drying, cells were hybridized with DNA probe (see immuno-FISH probe preparation below) for 30 sec at 85°C and incubated overnight at 37°C.

The immuno-FISH probe was prepared by nick translation from the lacO-I-SceI plasmid that was used to create the I-Hela111 cell line. DNA probe (0.3 μg) was mixed with 9 μg of ssDNA and 3 μg of CotI human DNA (Roche) and precipitated with 2.5× vol of ethanol and 1/10 vol of 2.5 M sodium acetate for 30 min at −80°C. After 20 min of centrifugation, the supernatant was discarded, and the pellet was washed with 70% ethanol and centrifuged again for 5 min. The supernatant was discarded, and the pellet was dried. The pellet was resuspended in 20 μL of hybridization solution (50% formamide, 4× SSC, 10% dextran sulfate) per coverslip by vortexing for 1 h. The probe was denaturated for 5 min at 90°C and preannealed for at least 15 min at 37°C before hybridization with cells.

The day after hybridization, immuno-FISH was revealed. Coverslips were washed twice for 20 min at 42°C in 2× SSC and then incubated with secondary antibody and fluorescein anti-biotin (Vector Laboratories, SP-3040) at 1:100 dilution for 45 min. Coverslips were washed, incubated with DAPI, and mounted in Prolong Gold reagent (Molecular Probes).

Slides were observed, and colocalization counting was done in epifluorescence microscopy. Pictures were taken with confocal microscopy. For experiments with Pom121-GFP-lacI constructs, cells were always costained with laminB to evaluate relocalization of the lacO array at the nuclear pores. For experiments with BRG1-cherry-lacI or cherry-lacI transfections, colocalization was counted using confocal microscopy.

### Time-lapse microscopy

Three-dimensional stacks were captured every 10 min for a total of 320 min upon NCS addition using the Leica DM6000 microscope with Leica CSU22 spinning disc and Andor Ixon 897 camera. Twenty different cells were imaged for each condition (±NCS).

## Supplementary Material

Supplemental Material
