# Supplementary material for: Nuclear position dictates DNA repair pathway choice
Source: Genes Dev. 2014 Nov 15;28(22):2450–63. doi: 10.1101/gad.248369.114 (PMC4233239; doi:10.1101/gad.248369.114)
Supplement: Supplemental Material [file supp_28_22_2450__index.html]

Nuclear position dictates DNA repair pathway choice — Supplemental Material 

# Nuclear position dictates DNA repair pathway choice

## Supplemental Material

**Files in this Data Supplement:**

- Supp Material.pdf
