# Supplementary material for: Nuclear position dictates DNA repair pathway choice
Source: Genes Dev. 2014 Nov 15;28(22):2450–63. doi: 10.1101/gad.248369.114 (PMC4233239; doi:10.1101/gad.248369.114)

Materials and methods

Cloning and plasmids

**Construct**	**Origin**
GFP-lacI-ΔEMD	(Reddy et al. 2008)
GFP-lacI	(Reddy et al. 2008)
cherry-lacI	(Soutoglou and Misteli 2008)
BRG1- cherry-lacI	gift from Tom Misteli
Pom121 GFP lacI	(see below)
pEXPR-EF1α-Pom121A-Venus	gift from Naoko Imamoto
pWHE320-HA-I-SceI	(Lemaitre et al. 2012)
pWHE146-Tet activator	(Lemaitre et al. 2012)

pCXPA-POM121A-EGFP-LacI was assembled with the universal expression system (manuscript in preparation) in a single cloning reaction with 5 fragments and using type IIS restriction enzymes

Super-resolution imaging and analysis

Immunofluorescence was performed as described above. Postfixation in 4% formaldehyde for 20 min was performed prior to imaging. The super-resolution microscopy experiments were performed on a Leica SR GSD system that consists of: Leica DMI6000 B inverted microscope with HCX PL APO 100x/1.47 Oil CORR TIRF PIFOC objective and 1.6x magnification lens for resulting pixel size of 100 nm; Andor

iXon3 DU-897U-CS0-#BV EMCCD camera with field of view of 18x18 μm in GSDIM mode; continuous wave fibre lasers (MPBC Inc., 488 nm 300 mW, 532 nm 1000 mW, 642 nm 500 mW); a diode laser 405 nm 30 mW; supressed motion (SuMo) sample stage with reduced drift.

For the super-resolution imaging the samples were mounted in PBS buffer that contained 10 mM of cysteamine (Sigma) and that was adjusted to pH 7.5 with 25 mM of HEPES. MEA was dissolved at 1M in PBS and was stored at -20°C. The final dilution was done prior to imaging.

For imaging of Alexa-488 we used the 488 nm laser as excitation source, filter cube with excitation filter DBP 405/10 488/10, dichroic mirror LP 496 and emission filter BP 555/100. For Alexa-647 – 642 nm laser, DBP 405/10 642/10, LP 649 and BP 710/100, respectively. The two colour channels were imaged sequentially: first Alexa-647, then Alexa-488. The excitations were performed at 100% power of corresponding lasers; the acquisitions started after beginning of observation of single-fluorophore events ("blinking") that corresponded to 1-2 min of excitation for Alexa-488 and 1-5 s for Alexa-647. The time of exposition of a frame was 50 ms at 488 nm and 10 ms at 647 nm. After few minutes of acquisition, as number of blinking evens dropped, the sample started to be illuminated additionally by 405 nm laser with gradual increase of its intensity in order to keep a constant rate of single-molecular returns into the ground state. The acquisition stopped after complete bleaching of the fluorophore. Duration of acquisitions was typically 10-20 min for Alexa-488 and 7-10 min for Alexa-647.

The localization and fitting of single-molecular events were performed in Leica LAS AF 3.2.0.9652 software with "center of mass" fitting method. Close events on consecutive frames, most likely originating from the same fluorophore, were merged using a corresponding option in the software. Maximal number of events to merge was set to 10, radius – to 50 nm. The obtained event lists, containing for each event: frame ID, coordinates x y, fitted number of photons, standard deviations σ_x σ_y for fitted 2D-Gaussians, were exported in an .ascii file and analysed further using a custom software written in Matlab. Super-resolution images, were calclulated with grey value of a pixel as quantity of localizations detected in the pixel area.

In order to reduce chromatic aberrations, the microscope was calibrated with multi-colour fluorescent beads (Tetraspeck, d=200 nm). The same area of a coverslip with beads was excited by 488 nm and 642 nm laser light; obtained pair of images appeared shifted on 20-60 nm for each bead, depending on lateral position of the bead in the field of view. The values of the offset were fitted to the x and y position on the image by a 2-order polynom. The obtained fit was subtracted from coordinates of each event of the red channel, resulting in residual chromatic offset less than 25 nm through all the field of view.

In order to reduce a drift of the sample, each single-color acquisition was divided onto two successive parts with equal number of events. From each part, a super-resolution image was reconstructed. The shift between the two images was calculated with subpixel precision by cross-correlation using a Matlab function(Manuel Guizar-Sicairos 2008) . The obtained value was fitted linearly into full range of frames and was subtracted from each single-molecular localization. The red channel events were shifted towards the final frame of the red colour acquisition,

the green channel ones – towards the first frame of the green colour acquisition, in order to reduce an additional offset between two colours, produced by drift and sequential imaging.

We were not able to reliably calculate shifts between smaller datasets due to not enough quantity of localizations for reconstruction more than two resembling images. So with this approach only a constant component of drift may be reduced, that is yet the most significant on our system.

We performed coordinate-based colocalization analysis of single-molecule localization data of two species. For each single-molecular event A*i* we calculated a colocalization value C_{Ai} that adapts values from -1 (for anti-correlated distributions) through 0 (for non-correlated) to +1 (for perfectly correlated distributions)(Malkusch et al. 2012). For the calculation of C_{Ai} we took into account all the localizations of both colours around A*i* within radii from 2 nm to 500 nm with step of 2 nm.

A histogram of distribution of C_A showing overall colocalization level was calculated for each double-colour image. We also calculated a global colocalization value for each image by division the sum of all positive values C_{Ai} by the sum of all negative values.

Quantification of the distribution of m6A-Tracer intensity

The quantification of distribution of m6a-Tracer was done using a macro on ImageJ, available upon request. Ratio of intensity of m6a-Tracer in the nucleoplasm over the intensity at the nuclear enveloped was then calculated.

Antibodies

Antibodies	Company	Reference	Application
laminB	Santa Cruz	SC-6216	ImmunoFISH, IF
γH2AX	Abcam	Ab22551	ImmunoFISH, IF
53BP1	Novus	NB100-304	ImmunoFISH, IF
Brca1	Calbiochem	OP92+OP93	ImmunoFISH, IF
Rad51	Calbiochem	PC130	ImmunoFISH, ChIP
Rad54	Abcam	Ab11055	ImmunoFISH
Ku80	Santa Cruz	SC-56136	ImmunoFISH
γH2AX	Abcam	Ab2893	ChIP
RPA	Novus	NB600-565	ChIP
P-RPA	Bethyl	A-300 245A	ChIP
BRCA1	Santa Cruz	SC-642	ChIP
XRCC4	Abcam	Ab145	ChIP, WB
Tubulin	Sigma	DM1A	WB
GFP	IGBMC		IF prior to GSDIM
TPR	Abcam	Ab84516	IF prior to GSDIM

Electron spectroscopic imaging (ESI) of chromatin structure and variation. Human U2OS osteosarcoma cells were treated with either vehicle (0.1% DMSO) or with 500 nM trichostatin A (TSA) for 4 h before being fixed in 4% paraformaldehyde (EMS) for 10 min at room temperature (RT) prior to being permeabilized in PBS containing 0.5% Triton X-100 for 5 min. Cells were then “post fixed” in 1% glutaraldehyde

(EMS) for 5 min at RT to maintain chromatin structure during resin embedding. The cells were then dehydrated in an ethanol series and embedded in Quetol 651 (EMS) before being processed, sectioned and imaged by ESI as previously described (Dellaire G 2004)using a Tecnai 20 transmission electron microscope (FEI) equipped with an energy-filtering spectrometer (Gatan). Energy-filtered electron micrographs of nitrogen (N) and phosphorus (P) were collected, and non-chromosomal protein was segmented by subtracting the N from the P ESI micrograph, which was then false colored in cyan and combined in a composite image with the P ESI micrograph false colored in yellow in Photoshop CS6 (Adobe) to highlight chromatin. The composite elemental maps of N-P (cyan) and P (yellow) were then analyzed for thickness of nuclear-lamina-associated chromatin using Image J v1.48k software (NIH). Pixel measurements (50 measurements taken from 10 cells) were converted into microns (μm) and then averaged per cell, and the data was represented as mean chromatin thickness± SEM (where N=10). Statistical significance between cell lines was generated using the Student's *t* test in Excel (Microsoft). The mean coefficient of variation (CV) in chromatin density was calculated for chromatin within the nucleus of vehicle and TSA treated U2OS cells (N=5), using phosphorus-enriched 155 keV electron micrographs as previously described(Dellaire et al. 2009). Briefly, the mean and SD pixel intensities were first determined from 5 X 10 pixel-wide line scans per cell using Image J. Then for each cell the CV was determined by dividing the mean pixel intensity by the SD, after which the CVs were averaged for vehicle or TSA treated cells and represented as a percentage ± SEM.

Measurement of the size of the lacO array

The lacO array sizes at different conditions were measured on paraformaldehyde fixed samples. The images were taken by Leica DM6000 microscope with Leica CSU22 spinning disc and Andor Ixon 897 camera. For every condition at least 20 individual cells were imaged and analyzed. The Z planes were taken every 0.3 mm. For 3D reconstruction and quantification of volumes the Imaris software (Bitplane) was used.

ChromatinIP

The ChIP analysis was done following the Dynabeads ChIP protocol from Abcam(Pankotai et al. 2012) with a few modifications. Briefly, one 150-mm dish with cells that were 70% confluent was used for each time point. The cells were cross-linked for 30 min in 0.75% (v/v) paraformaldehyde and then sonicated in 1% (v/v) SDS-containing sonication buffer (50 mM HEPES, pH 8, 140 mM NaCl, 1 mM EDTA, 1% (v/v) TritonX-100, 1% (v/v) SDS and a protease inhibitor cocktail (Roche)). Thirty milligrams of chromatin were diluted in RIPA buffer (50 mM Tris-HCl, pH 8, 150 mM NaCl, 1 mM EDTA, 1% Triton X-100 (v/v), 0.1% sodium deoxycholate (w/v) and 0.1% SDS (v/v)) and were used in each immunoprecipitation by adding 4 µg of antibody and 50 µl Dynabeads M-280 (Invitrogen). The beads were washed for 5 min with low-salt buffer (20 mM Tris-HCl, pH 8, 150 mM NaCl, 2 mM EDTA, 1% (v/v) Triton X-100 and 0.1% (v/v) SDS), then 5 min with high-salt buffer (20 mM Tris-HCl, pH 8, 500 mM NaCl, 2 mM EDTA, 1% (v/v) Triton X-100 and 0.1% (v/v) SDS) and for 5 min with LiCl buffer (10 mM Tris-HCl, pH 8, 250 mM LiCl, 1 mM EDTA, 1% (v/v) NP-40 and 1% (w/v) sodium deoxycholate) and two times for 5 min with TE

buffer. The elution was done twice at 65 °C for 15 min. Cross-links were reversed by incubation at 65 °C for 6 h. The DNA was purified after proteinase K and RNaseA treatment by using phenol-chloroform extraction and was resuspended in 50 μl of TE buffer.

The signal in each experiment was calculated using the formula (immunoprecipitated sample–IgG control)/input, and each value represents a relative DNA concentration that is based on the standard curve of the input.

Dellaire G, Kepkay R, Bazett-Jones DP. 2009. High resolution imaging of changes in the structure and spatial organization of chromatin, γ-H2A.X and the MRN complex within etoposide-induced DNA repair foci. *Cell Cycle* **8**: 3750-3769.

Dellaire G NR, and Bazett-Jones DP 2004. Correlative light and electron spectroscopic imaging of chromatin in situ. *Methods Enzymol* **375**: 456-478.

Malkusch S, Endesfelder U, Mondry J, Gelléri M, Verveer P, Heilemann M. 2012. Coordinate-based colocalization analysis of single-molecule localization microscopy data. *Histochem Cell Biol* **137**: 1-10.

Manuel Guizar-Sicairos STT, and James R. Fienup 2008. Efficient subpixel image registration algorithms. *Opt Letter* **33**: 156-158.

Supplementary figure legends

Figure S1- Experimental system

(A) Schematic representation of the experimental system: the lac repressor (GFP-lacI) binds to the lac operator (lacO), which allows the relocalization of the lacO array to the nuclear lamina when fused to ΔEMD. The addition of Dox allows the expression of ISceI and induction of a DSB at the I-SceI restriction site, which is located next to the lacO repeats. **(B)** Quantification of the colocalization of the lacO array with lamin B in absence or presence of dox for 14h. Values represent means ± SD from three independent experiments (number of cells analyzed per experiment ≥ 50). **(C)** 3D Z-stacks confocal microscopy images of the lacO array (green) and lamin B (gray) in I-U2OS19 GFP-lacI cells (upper panel) or GFP-lacI-ΔEMD cells (lower panel) **(D)** LM-PCR in GFP-lacI and GFP-lacI- ΔEMD cells 14h after Dox addition. Products are shown (upper panel) and quantified (below) after 26 (left) or 28 (right) PCR cycles.

Figure S2- DDR is delayed at the nuclear lamina

(A) Quantification of the colocalization of the lacO array with γH2AX at the indicated times after dox addition in I-HeLa111 infected with GFP-lacI or GFP-lacI-ΔEMD. Values represent means ± SD of three independent experiments (number of cells analyzed per experiment ≥ 50). (B) Immuno-FISH single-Z confocal images of the lacO array (green) and γH2AX (red) in I-HeLa111 cells expressing GFP-lacI or GFP-lacI-ΔEMD and treated or not with Dox for 14h. For statistical analysis, t test was performed. P value are represented as follows : *<0.05, **<0.01

Figure S3- The expression of GFP-lacI-ΔEMD does not impair DDR activation

(A) Immuno-FISH single-Z confocal images of the lacO array (green) and lamin B (red) in I-U2OS19 GFP-lacI cells (left) or GFP-lacI-ΔEMD cells (right) in presence of 2mM IPTG and in the absence (upper panel) or presence (lower panel) of Dox for 14h. **(B)** Single-Z confocal microscopy images of the lacO array (green) and γH2AX (red) in I-U2OS19 GFP-lacI cells (left) or GFP-lacI-ΔEMD cells (right) in presence of 2mM IPTG and in the absence (upper panel) or presence (lower panel) of Dox for 14h, after immuno-FISH. **(C)** Percentage of the colocalization of the lacO array with lamin B in presence of 2mM IPTG, and in absence or presence of doxycycline for 14h. **(D)** Quantification of the colocalization of the lacO array with γH2AX in presence of 2mM IPTG, and in absence or presence of Dox for 14h. Values represent means ± SD of three independent experiments (number of cells analyzed per experiment ≥ 50).

Figure S4-Recruitment of DSB repair factors at the nuclear lamina in I-U2OS19

Immuno-FISH single-Z confocal images of the lacO array (green), laminB (gray) and (A) Ku80 (red), (B) BRCA1 (red), (C) Rad51 (red) in I-U2OS19 cells expressing GFP-lacI or GFP-lacI-ΔEMD and treated or not with Dox for 14 or 20h

Figure S5-Recruitment of DSB repair factors at the nuclear lamina in I-HeLa111

Immuno-FISH single-Z confocal images of the lacO array (green), laminB (gray) and (A) Ku80 (red), (B) BRCA1 (red), (C) Rad51 (red) in I-HeLa111 cells expressing GFP-lacI or GFP-lacI-ΔEMD and treated or not with Dox for 14 or 20h **(D-F)** Quantification of the colocalization of the lacO array with **(D)** Ku80, **(E)** BRCA1, **(F)** RAD51, at the indicated times after dox addition in I-HeLa111 infected with GFP-lacI or GFP-lacI-ΔEMD. Values represent means ± SD of three independent experiments (number of cells analyzed per experiment ≥ 50). For statistical analysis, t test was performed. P value are represented as follows : *<0.05, **<0.01

Figure S6-Recruitment of Rad54 at the nuclear lamina in I-U2OS19

(A) Quantification of the colocalization of the lacO array with Rad54 at the indicated times upon Dox addition in I-U2OS19 GFP-lacI or GFP-lacI-ΔEMD cells. Values represent means ± SD of three independent experiments (number of cells analyzed per experiment ≥ 50). For statistical analysis, t test was performed. P value are

represented as follows : *<0.05 **(B)** Cell cycle profiles of I-U2OS19 GFP-lacI and I-U2OS19 GFP-lacI-ΔEMD cells.

Figure S7- TSA treatment induces chromatin decompaction

(A) Immunofluorescence images depicting H4acetylation (red) in I-U2OS19 GFP-lacI cells treated with DMSO or TSA. Nuclear stain, DAPI (blue) **(B-C)** Human U2OS osteosarcoma cells were treated with **(B)** vehicle (0.1% DMSO) or **(C)** with 500 nM trichostatin A (TSA) for 4 h before fixation and processing for electron spectrocopic imaging (ESI). In each row a low magnification phosphorus-enriched (155 KeV) electron micrograph is shown at the left, a line-scan of phosphorus intensity across the cell nucleus (between the white arrows) is shown in the middle panel, and on the far right a high magnification ESI electron micrograph is shown of the region outlined by a white dashed box in the low magnification micrograph. The coefficient of variation (CV) is also shown for the phosphorus intensity across the nuclei of vehicle and TSA treated cells (n=5; ± SEM); which represents the degree of variability in chromatin density as a percentage, where a lower percentage indicates a more homogenous chromatin density. The ESI micrographs have been false coloured such that chromatin appears yellow and non-chromosomal protein (e.g. nucleopores, marked by white astericks) appears cyan. The thickness of the nuclear lamina associate chromatin is demarcated by white arrow heads, N = nucleoli, and the scale bars = 1 micron. **(D)** The mean thickness of condensed chromatin associated with the nuclear lamina for cells treated with vehicle or with 500 nM TSA and depicted as a bar graph. Error bars = SEM, N=10. *p < 0.001

(E) Immuno-FISH single-Z confocal images of the lacO array (green) and lamin B (red) in I-U2OS19 GFP-lacI cells (left) or GFP-lacI-ΔEMD cells (right) in presence of DMSO (uper panel) or TSA (lower panel). **(F)** Quantification of the colocalization of the lacO array with lamin B in absence or presence of dox for 14h in cells treated with DMSO or TSA for 4h. Values represent means ± SD of three independent experiments (number of cells analyzed per experiment ≥ 50).

Figure S8- H2AX phosphorylation at the nuclear lamina is rescued upon TSA treatment

(A) Immuno-FISH single-Z confocal images of the lacO array (green) and γH2AX (red) in I-U2OS19 GFP-lacI cells (left) or GFP-lacI-ΔEMD cells (right) in presence of DMSO (uper panel) or TSA (lower panel).

Figure S9- HR factors recruitment at the nuclear lamina is rescued upon TSA treatment

Immuno-FISH single-Z confocal images of the lacO array (green) and (A) BRCA1 (red) or (B) RAD51 in I-U2OS19 GFP-lacI cells (left) or GFP-lacI-ΔEMD cells (right) in presence of DMSO (uper panel) or TSA (lower panel).

Figure S10- BRG1 tethering induces chromatin decondensation

(A) Schematic representation of the experimental system. The lac repressor (GFP lacI/cherry-lacI) binding to the lac operator (lacO) allows the relocalization of the

lacO array at the nuclear lamina when fused to ΔEMD. The expression of BRG1-cherry-lacI allows local decondensation of the lacO/I-SceI locus. The addition of Dox allows the expression of I-SceI and induction of a DSB at the I-SceI restriction site, next to the lacO repeats. **(B)** Images of 3D reconstruction of nuclei (blue) and the lacO array (red). **(C)** Quantification of the volume of the lacO array, normalized to the volume of the nucleus in GFP-lacI and GFP-lacI-ΔEMD cells expressing cherry–lacI or BRG1-cherry-lacI.

Figure S11- BRG1 tethering rescues HR factors recruitment at the nuclear lamina

Immunofluorescence single-Z confocal images of (A) BRCA1 (gray) or (B) RAD51 (gray) in I-U2OS19 cells expressing GFP-lacI or GFP-lacI-ΔEMD, transfected with cherry-lacI or BRG1-cherry-lacI (red) and treated or not with Dox for 20h.

Figure S12- Recruitment of DDR and HR factors are not impaired by tethering at the nuclear pores

(A) Schematic representation of the experimental system (left panel) for relocalization of the lacO locus to nuclear pores. Expression of Pom121-GFP-lacI allows the repositioning of the lacO locus to the nuclear pores. Immuno-FISH single-Z confocal image (right upper panel) of the lacO array colocalizing with lamin B in Pom121-GFP-lacI expressing cells. D-Storm picture of Pom121-GFP-lacI (green) and nucleoporin TPR (red) showing colocalization of the lacO array with the nucleoporin TPR (right lower panel). Immuno-FISH single-Z confocal images of the lacO array (green), laminB (gray) and (B) 53BP1 (red), (C) BRCA1 (red), (D) RAD51 (red) in I-U2OS19 cells expressing GFP-lacI or GFP-lacI-ΔEMD and treated or not with Dox for 20h.

Figure S13- Positional stability of LADs upon DNA damage

(A) Schematic representation of the experimental system. IPTG addition for 2h in GFP-lacI-ΔEMD after lacO repositioning to the periphery and DSB induction (with 14h dox treatment) allows the dissociation of the lacI from the lacO and a potential movement away from the nuclear lamina. **(B)** Percentage of colocalization of the lacO array with lamin B in absence or presence of dox (14h) in GFP-lacI- ΔEMD treated (for 2h) or not with IPTG. Values represent means ± SD of three independent experiments (number of cells analyzed per experiment ≥ 50). **(C)** Time lapse microscopy on HT1080 cells expressing Dam-laminB1 and m6a-Tracer (green) upon addition (or not) of 50ng/mL NCS for 15min. **(D)** D-STORM pictures of LADs colocalization with γH2AX (red) in HT1080 cells expressing Dam-laminB1 and m6a-Tracer (green) upon addition (or not) of 50ng/mL NCS for 15 min and release for 2 h.

Figure S14- Validation of silencing of ligase 3, XRCC4, RAD51, PARP1, XRCC1 by siRNA

(A) Western blot for tubulin, XRCC4, Rad51, PARP1, XRCC1 in I-U2OS19 GFP-lacI or GFP-lacI-ΔEMD treated with corresponding siRNAs. **(B)** LM-PCR in GFP-lacI and GFP-lacI-ΔEMD cells non-treated, 14h after Dox addition or 36h after a 14h Dox

pulse. Products are shown (upper panel) and quantified (below) after 28 PCR cycles. The intensity of the products depicted is normalized to the products of the non-treated samples. (C) Quantitative RT-PCR analysis of ligase *3* expression levels in I-U2OS19 GFP-lacI or GFP-lacI-ΔEMD cells treated with siRNA that targets a scramble sequence (purple lines) and ligase 3 sequences (blue lines). (D) Percent colocalization of the lacO array with γH2AX in untreated cells (NT) or after 14h of Dox (time point 0) and subsequent release for 24h in I-U2OS19 cells expressing GFP-lacI or GFP-lacI-ΔEMD and transfected with different ligase3-specific siRNAs (siLig3-6 or siLig3-7).

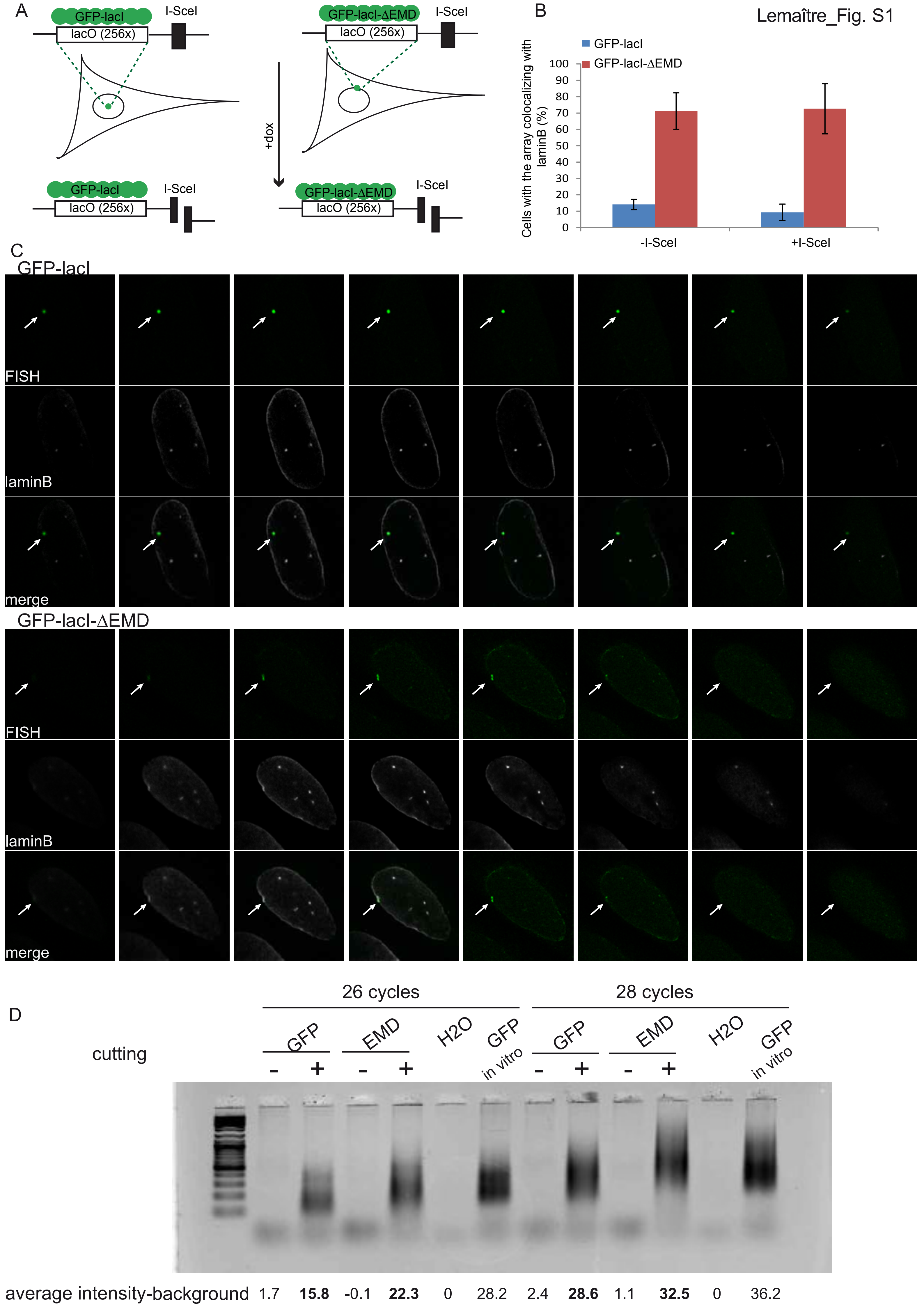
A
GFP-lacI
lacO (256x)
I-SceI
+dox
GFP-lacI-ΔEMD
B
GFP-lacI
GFP-lacI-ΔEMD
Cells with the array colocalizing with laminB (%)
-I-SceI
+I-SceI
C
GFP-lacI
FISH
laminB
merge
GFP-lacI-ΔEMD
D
26 cycles
28 cycles
cutting
GFP
EMD
H2O
GFP in vitro
average intensity-background 1.7 15.8 -0.1 22.3 0 28.2 2.4 28.6 1.1 32.5 0 36.2

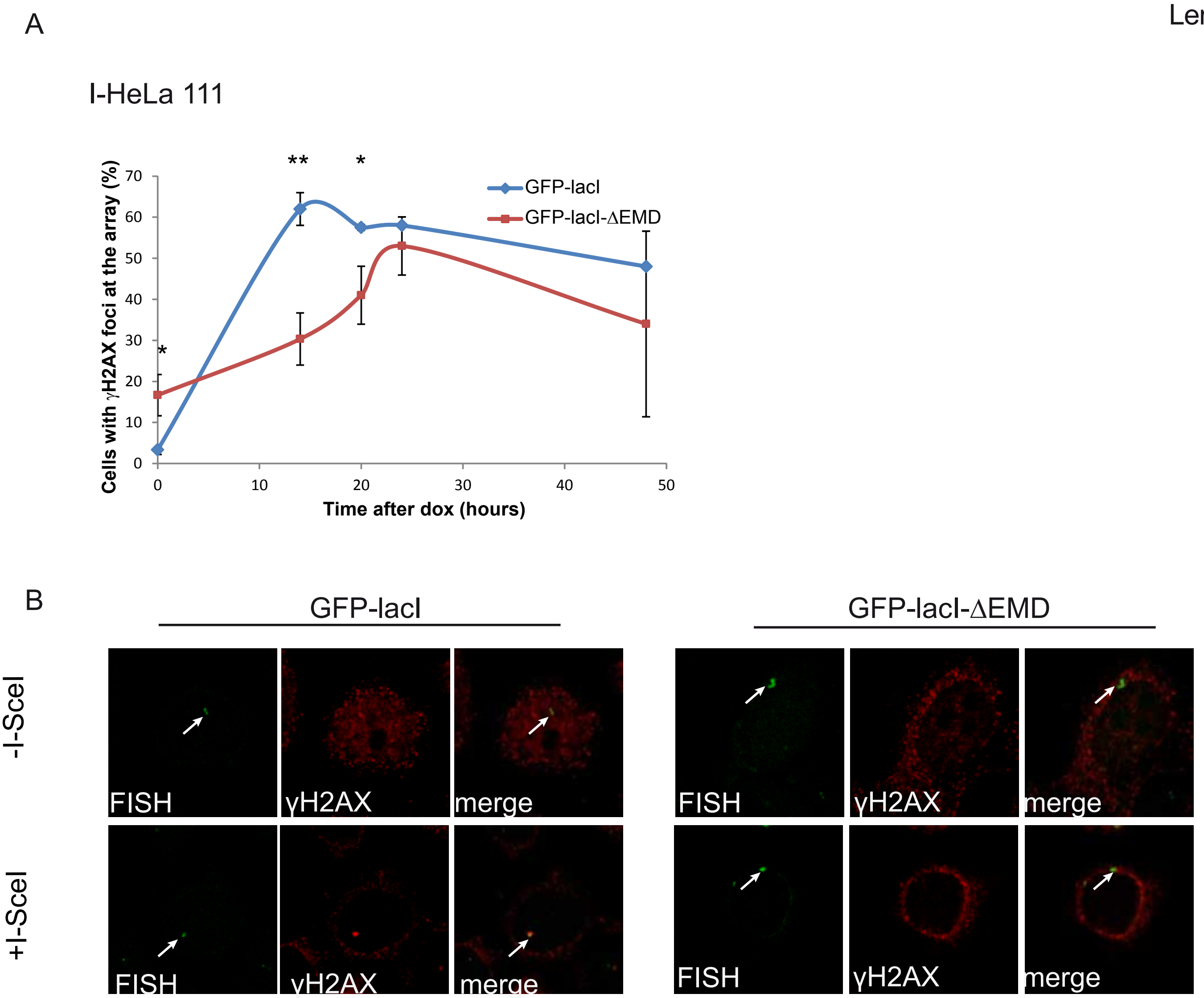
A
I-HeLa 111
**
*
*
GFP-lacI
GFP-lacI-ΔEMD
Cells with γH2AX foci at the array (%)
0
10
20
30
40
50
60
70
0
10
20
30
40
50
Time after dox (hours)
B
GFP-lacI
GFP-lacI-ΔEMD
-I-SceI
+I-SceI
FISH
γH2AX
merge
FISH
γH2AX
merge
FISH
γH2AX
merge
FISH
γH2AX
merge

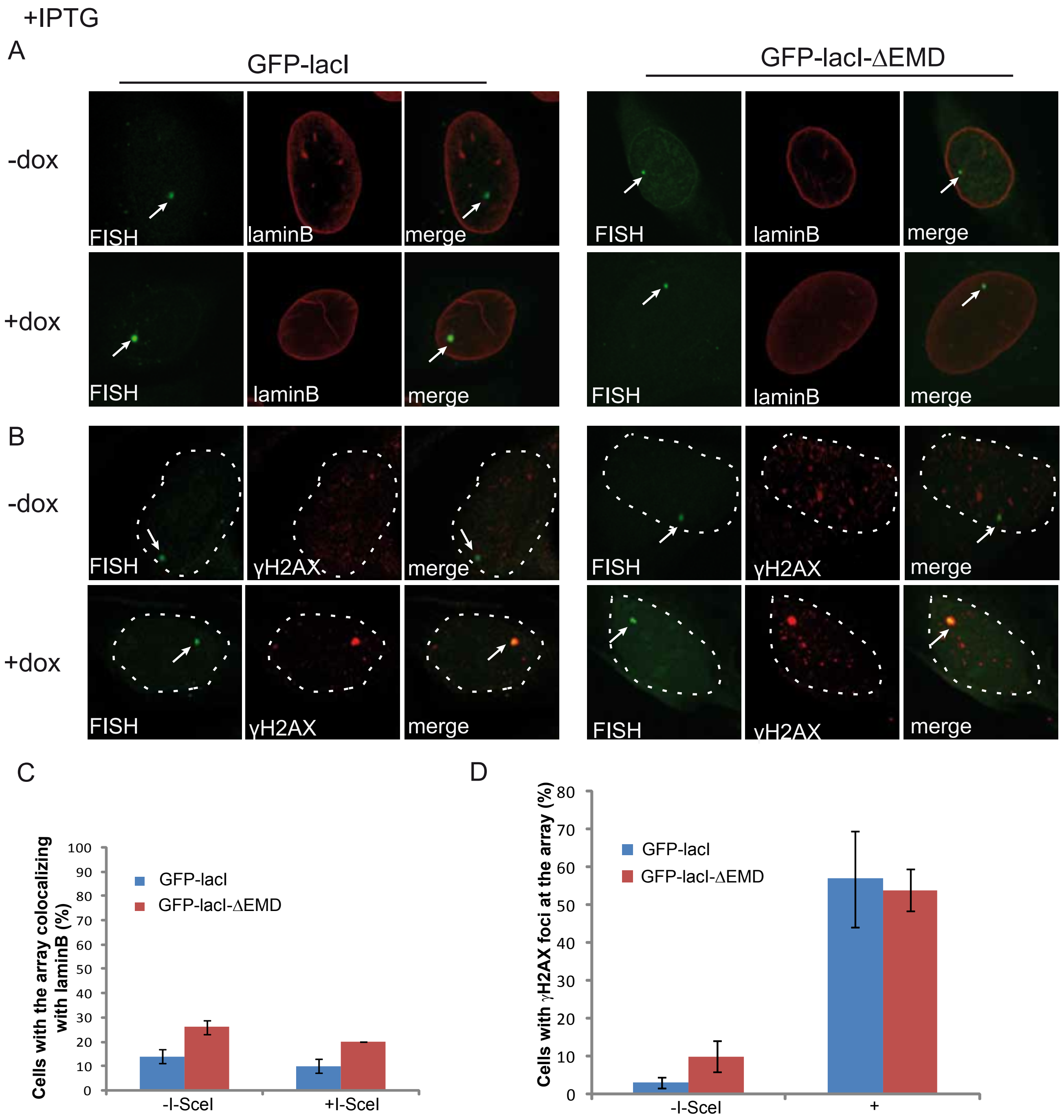
+IPTG
A
GFP-lacI
GFP-lacI-ΔEMD
-dox
+dox
FISH
laminB
merge
B
γH2AX
C
Cells with the array colocalizing with laminB (%)
100
90
80
70
60
50
40
30
20
10
0
GFP-lacI
GFP-lacI-ΔEMD
-I-SceI
+I-SceI
D
Cells with γH2AX foci at the array (%)
+

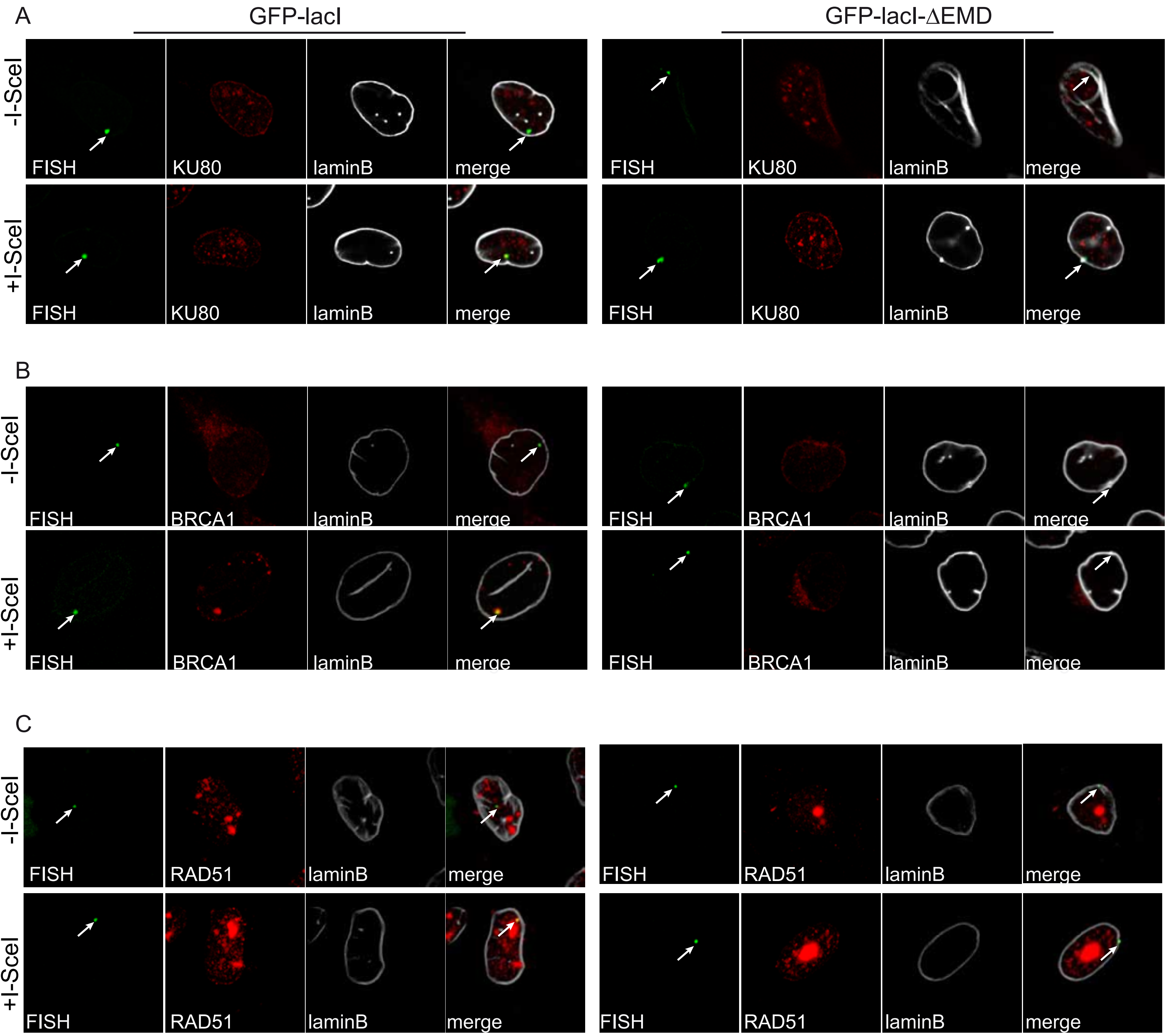
A
GFP-lacI
GFP-lacI-ΔEMD
-I-SceI
+I-SceI
FISH
KU80
laminB
merge
B
BRCA1
C
RAD51

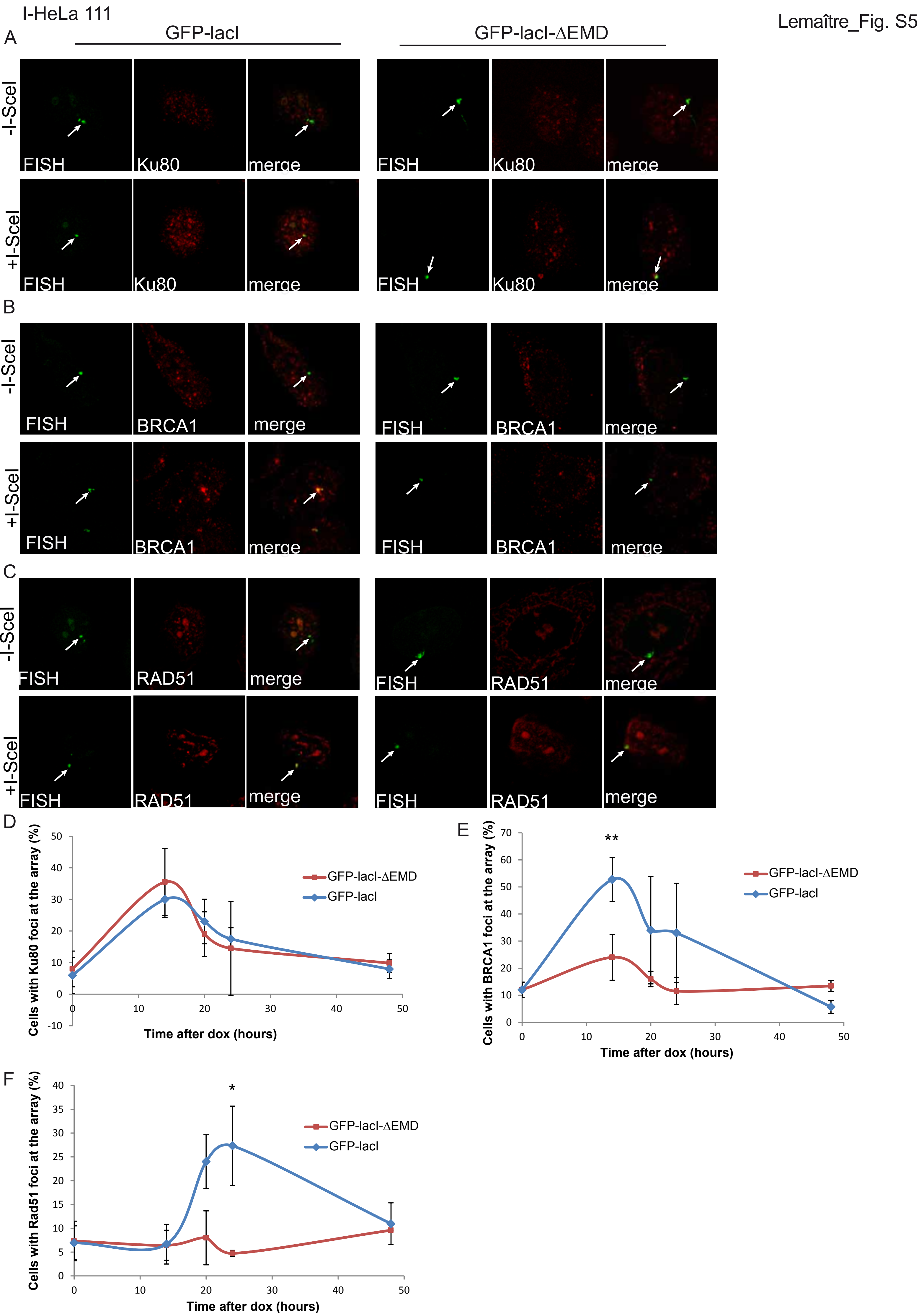

I-HeLa 111
A
GFP-lacI
GFP-lacI-ΔEMD
-I-SceI
+I-SceI
FISH
Ku80
merge
B
BRCA1
C
RAD51
D
Cells with Ku80 foci at the array (%)
Time after dox (hours)
GFP-lacI-ΔEMD
GFP-lacI
E
Cells with BRCA1 foci at the array (%)
**
F
Cells with Rad51 foci at the array (%)
*

I-U2OS19

A

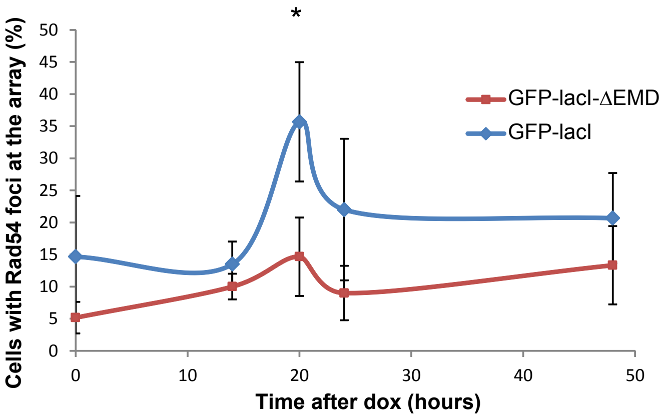
*
Cells with Rad54 foci at the array (%)
50
45
40
35
30
25
20
15
10
5
0
0
10
20
30
40
50
Time after dox (hours)
GFP-lacI-ΔEMD
GFP-lacI

B

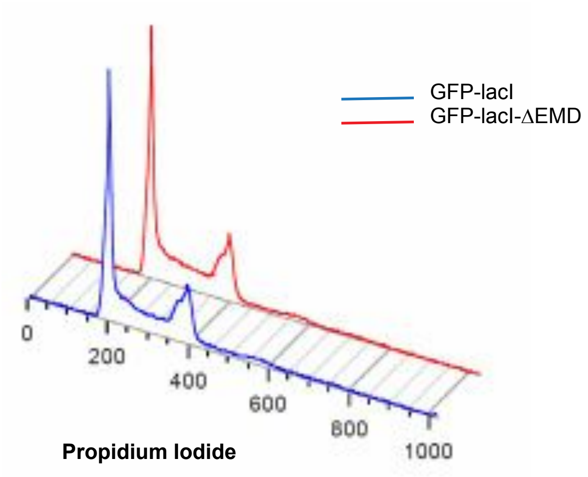
GFP-lacI
GFP-lacI-ΔEMD
0
200
400
600
800
1000
Propidium Iodide

Lemaître_Fig. S7

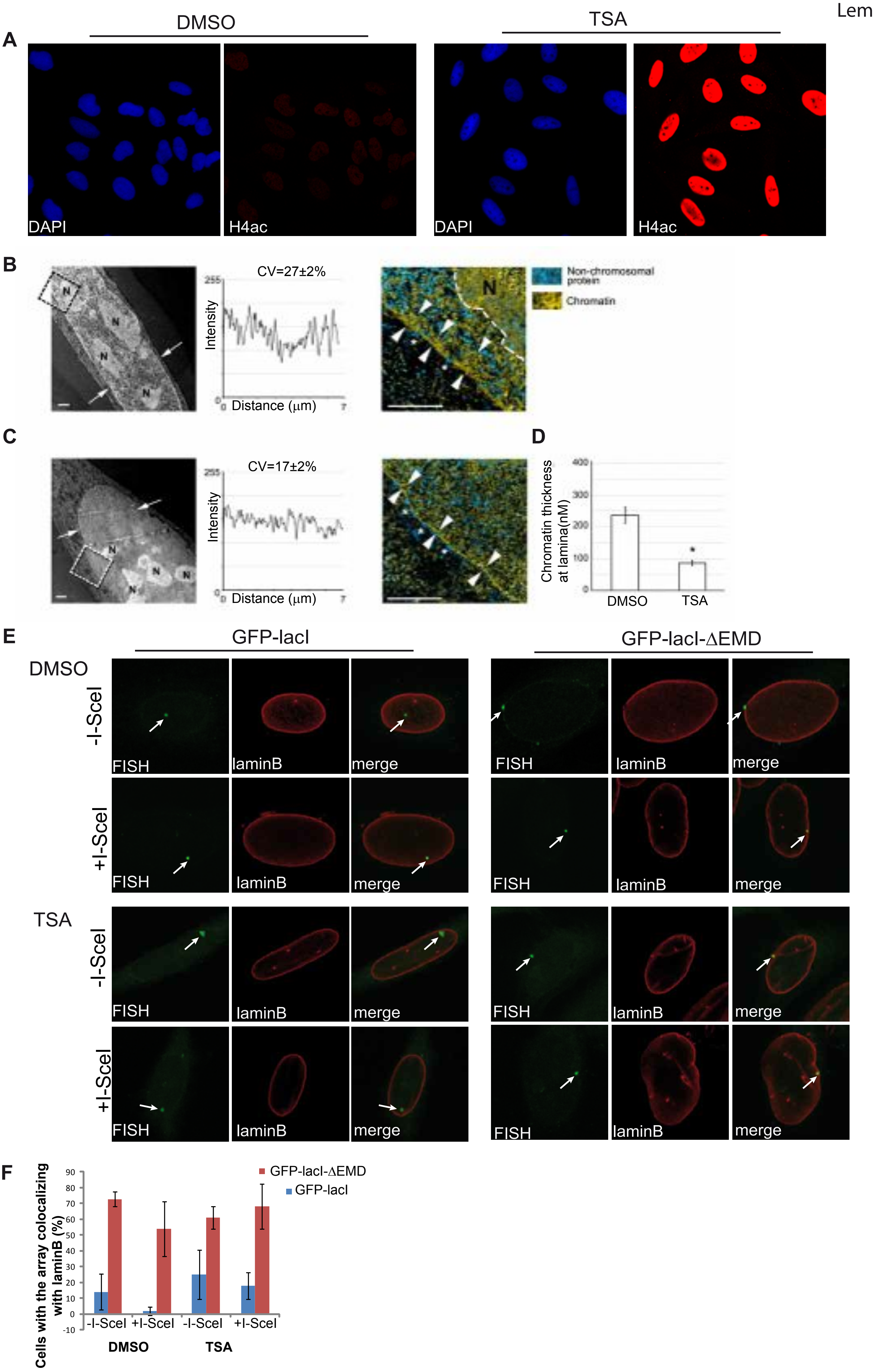

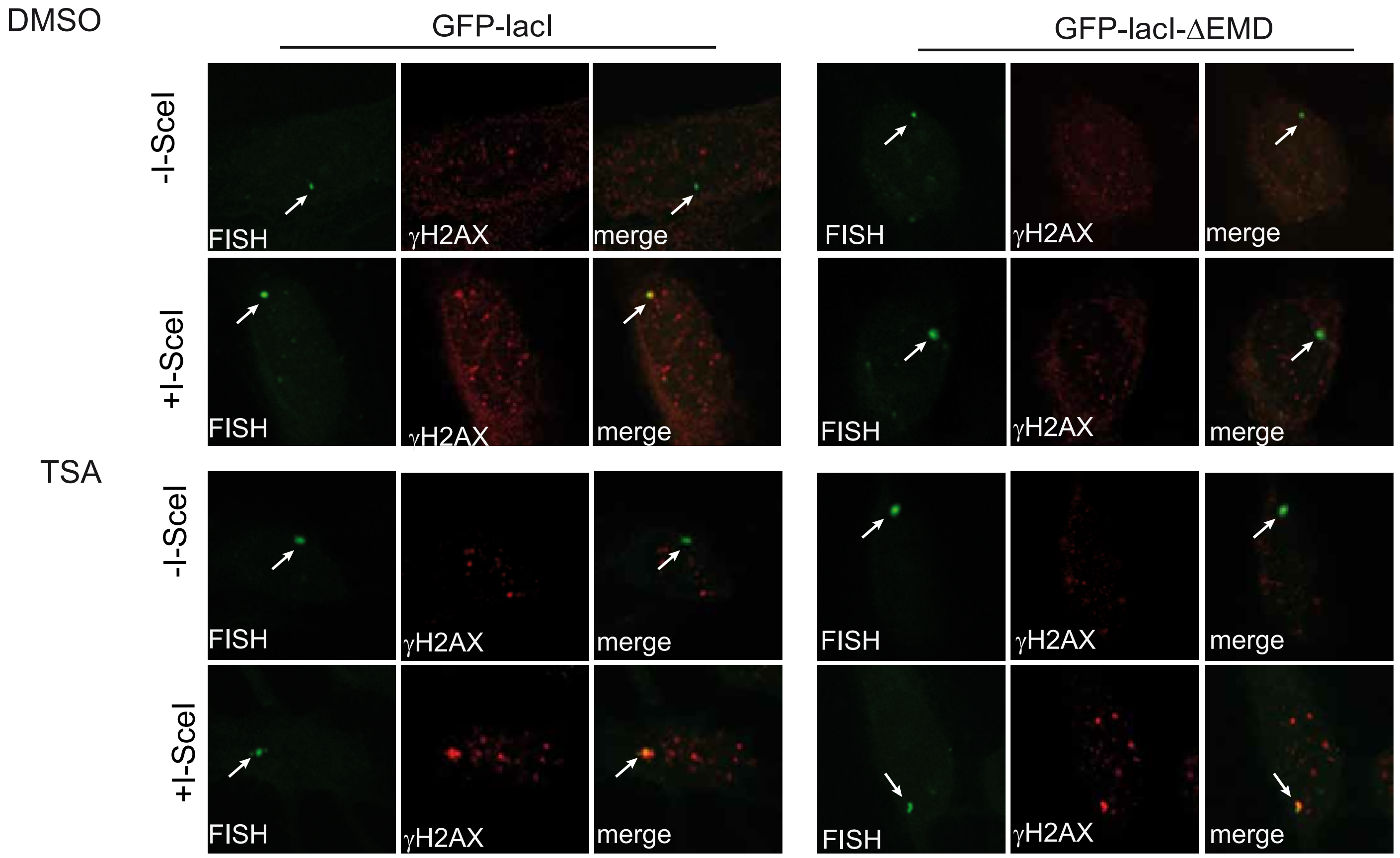
DMSO
GFP-lacI
GFP-lacI-ΔEMD
-I-SceI
+I-SceI
TSA
-I-SceI
+I-SceI
FISH
γH2AX
merge

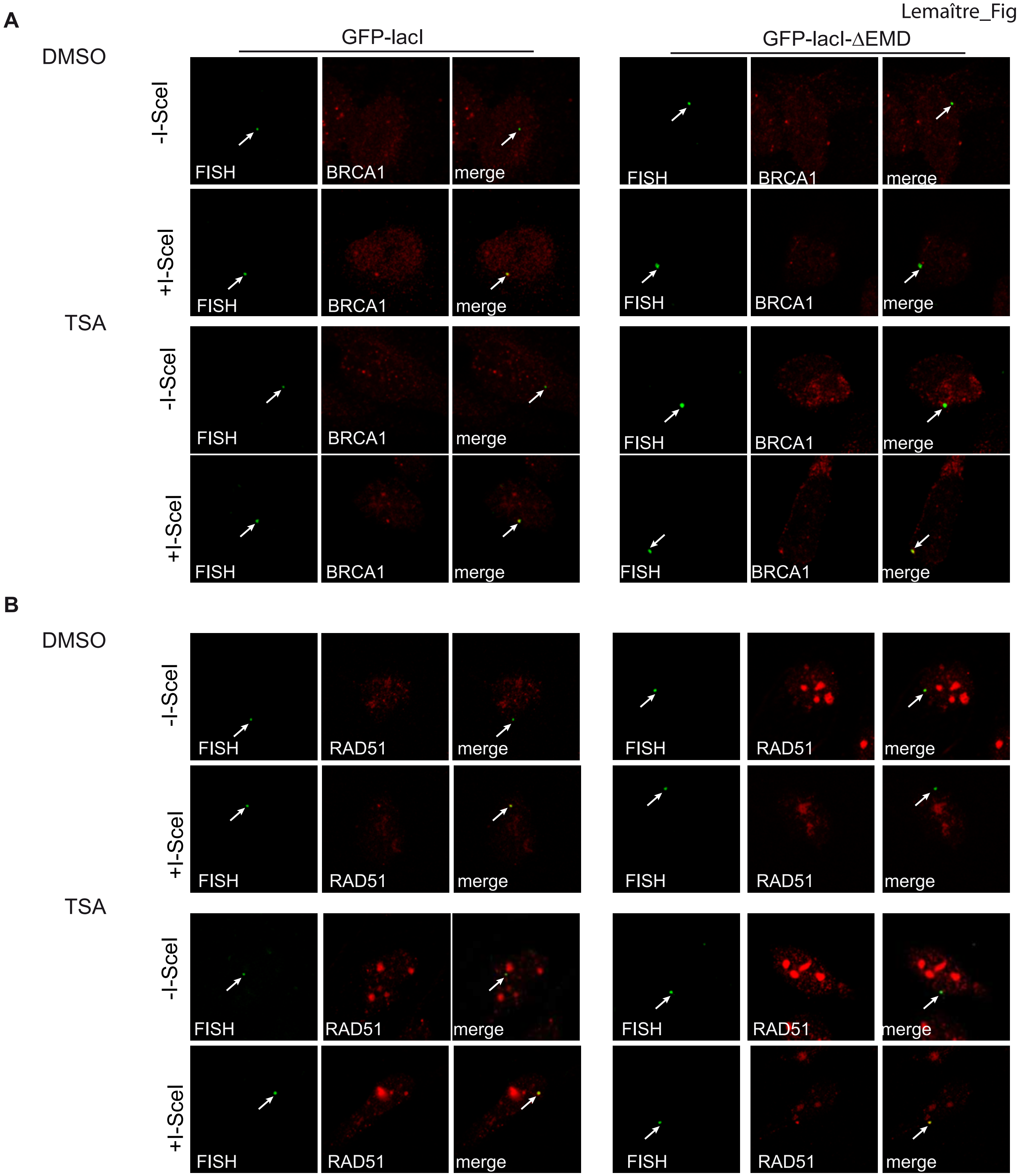
A
B
DMSO
TSA
GFP-lacI
GFP-lacI-ΔEMD
-I-SceI
+I-SceI
FISH
BRCA1
RAD51
merge

Lemaître_Fig. S10

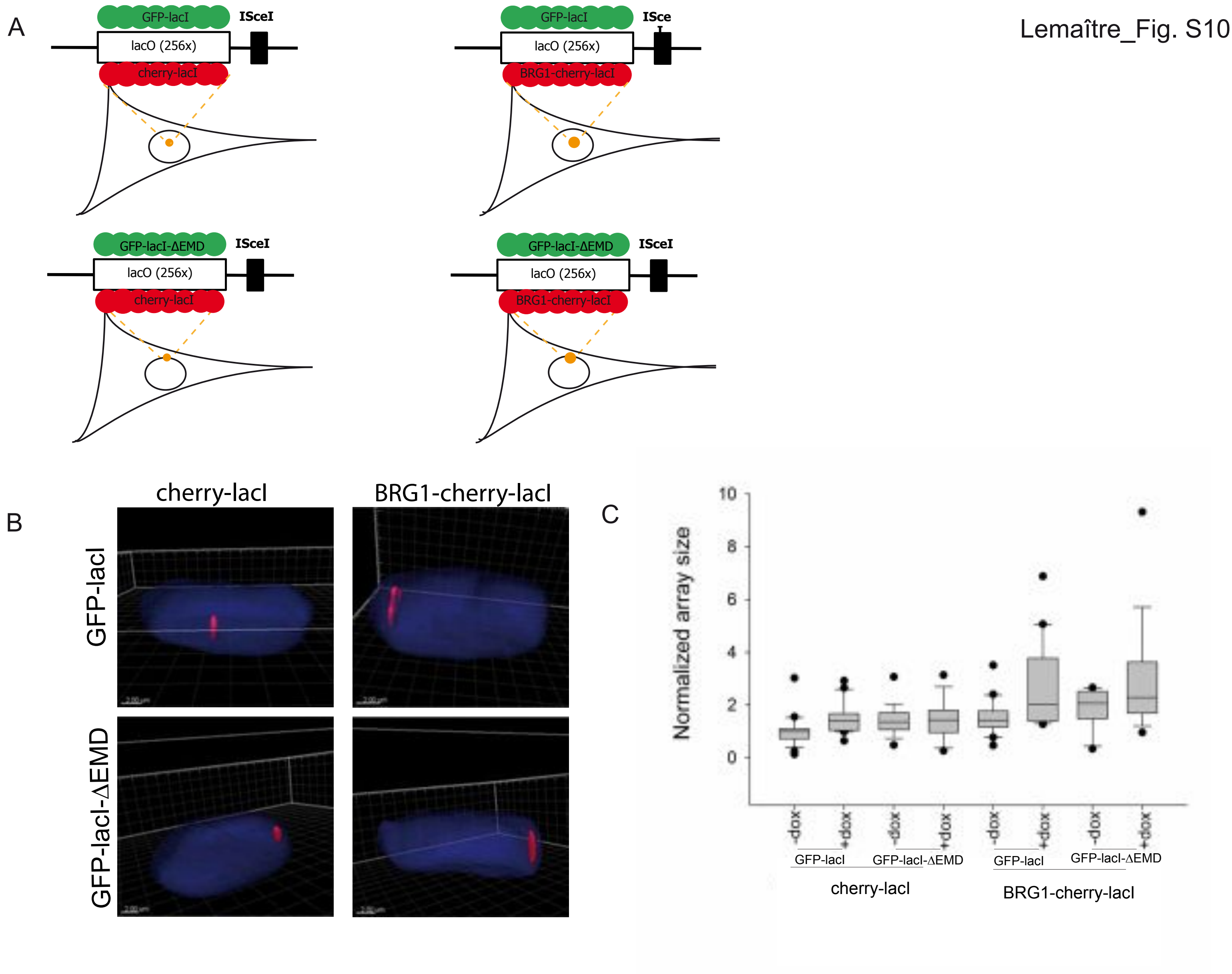

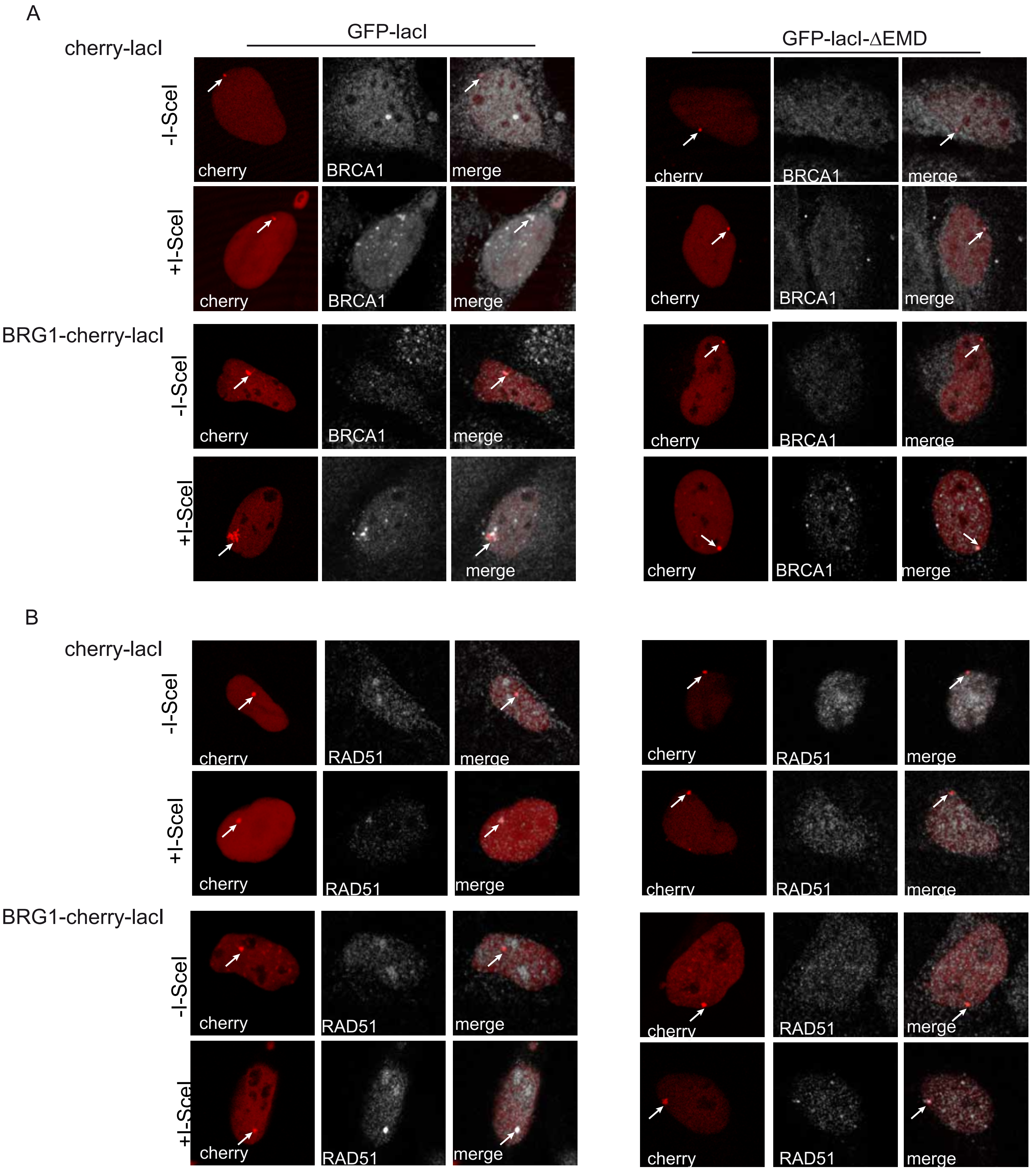
A
cherry-lacI
GFP-lacI
GFP-lacI-ΔEMD
-I-SceI
+I-SceI
BRG1-cherry-lacI
cherry
BRCA1
merge
B
RAD51

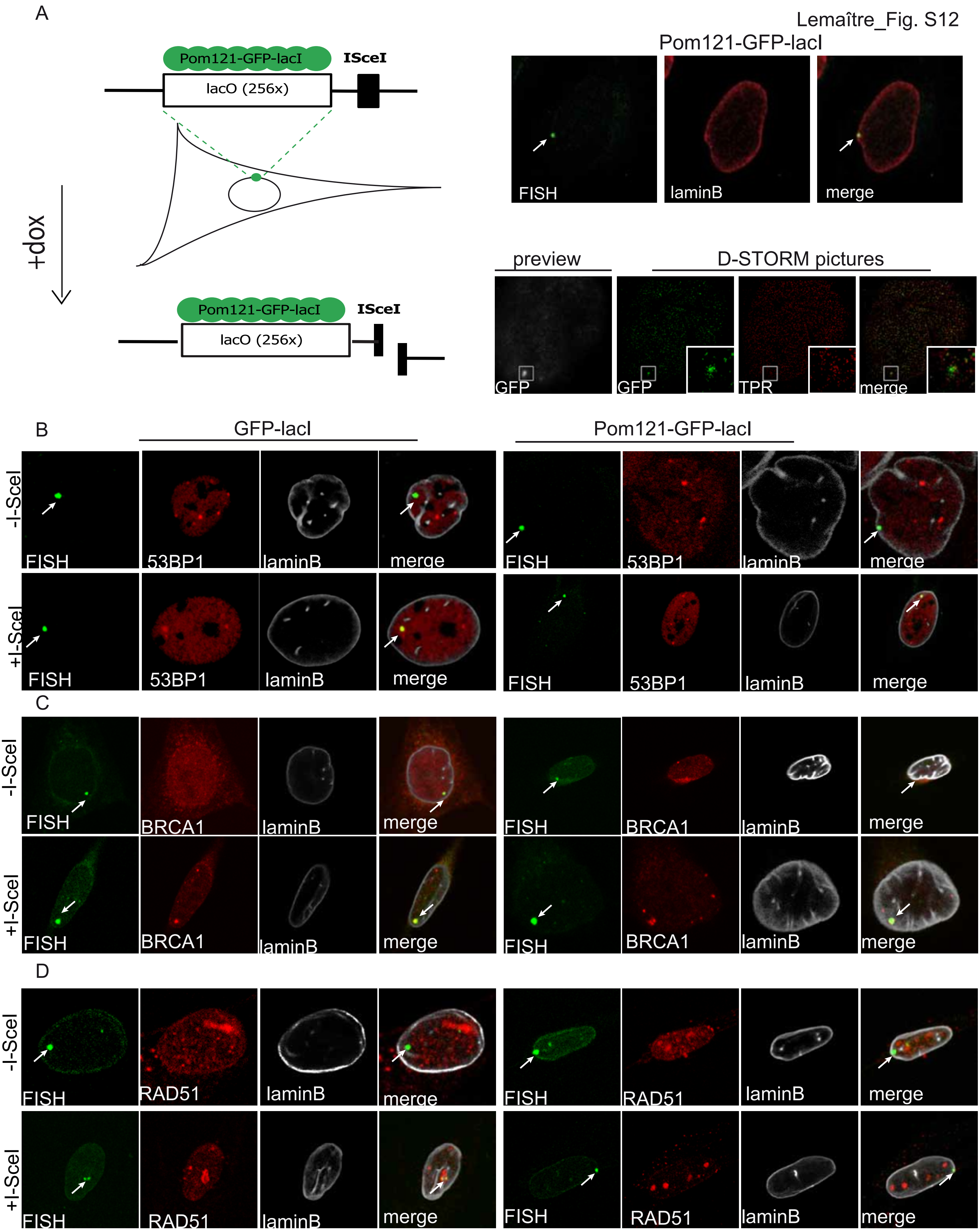
A
Pom121-GFP-lacI
ISceI
lacO (256x)
+dox
Pom121-GFP-lacI
ISceI
lacO (256x)
Pom121-GFP-lacI
FISH
laminB
merge
preview
D-STORM pictures
GFP
GFP
TPR
merge
B
GFP-lacI
Pom121-GFP-lacI
-I-SceI
+I-SceI
FISH
53BP1
laminB
merge
C
-I-SceI
+I-SceI
FISH
BRCA1
laminB
merge
D
-I-SceI
+I-SceI
FISH
RAD51
laminB
merge

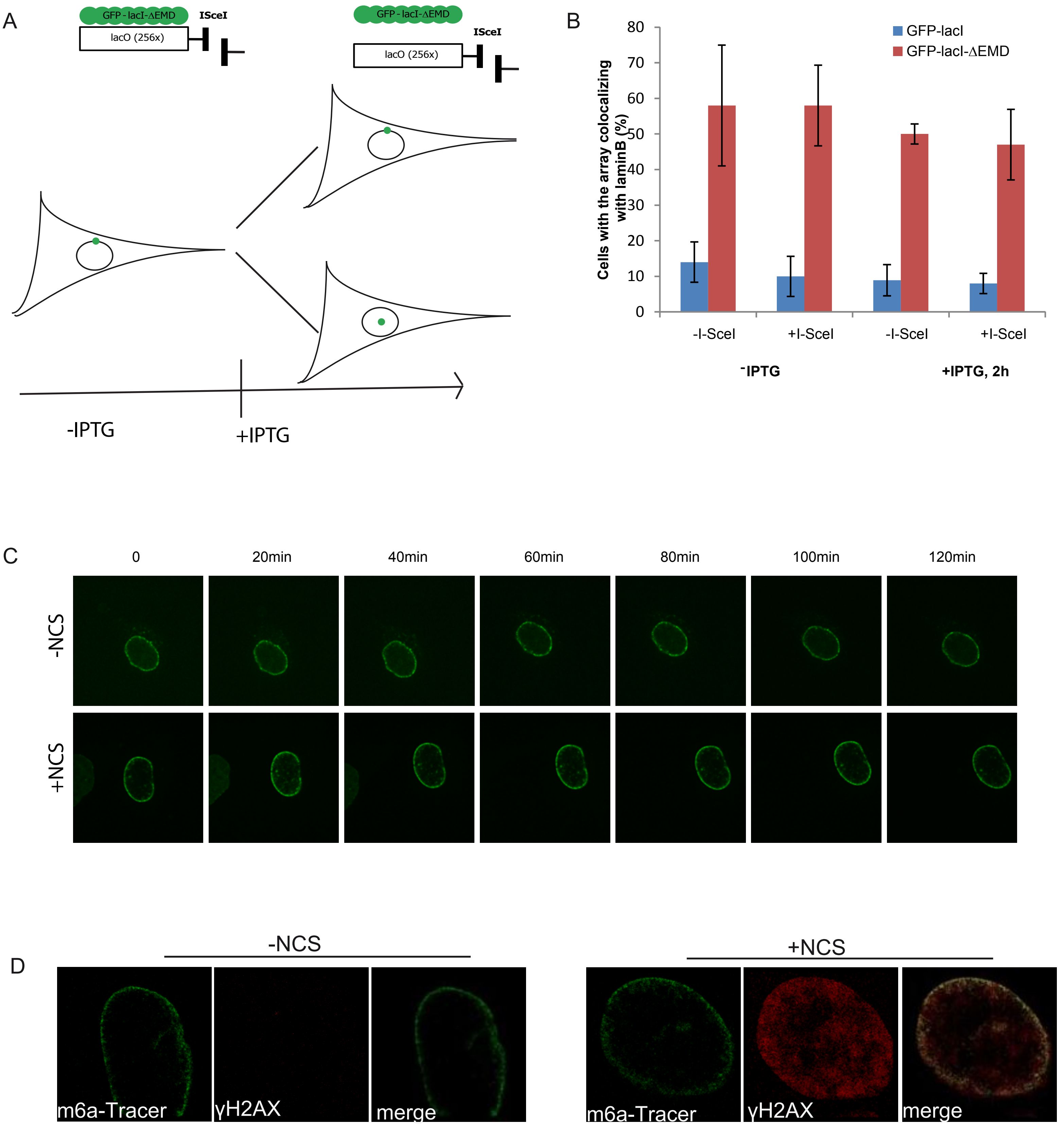
A
GFP - lacI-ΔEMD
IScel
lacO (256x)
GFP - lacI-ΔEMD
IScel
lacO (256x)
-IPTG
+IPTG
B
Cells with the array colocalizing with laminB (%)
80
70
60
50
40
30
20
10
0
GFP-lacI
GFP-lacI-ΔEMD
-I-SceI
+I-SceI
-I-SceI
+I-SceI
-IPTG
+IPTG, 2h
C
0
20min
40min
60min
80min
100min
120min
-NCS
+NCS
D
-NCS
+NCS
m6a-Tracer
γH2AX
merge
m6a-Tracer
γH2AX
merge

Lemaître_Fig. S14

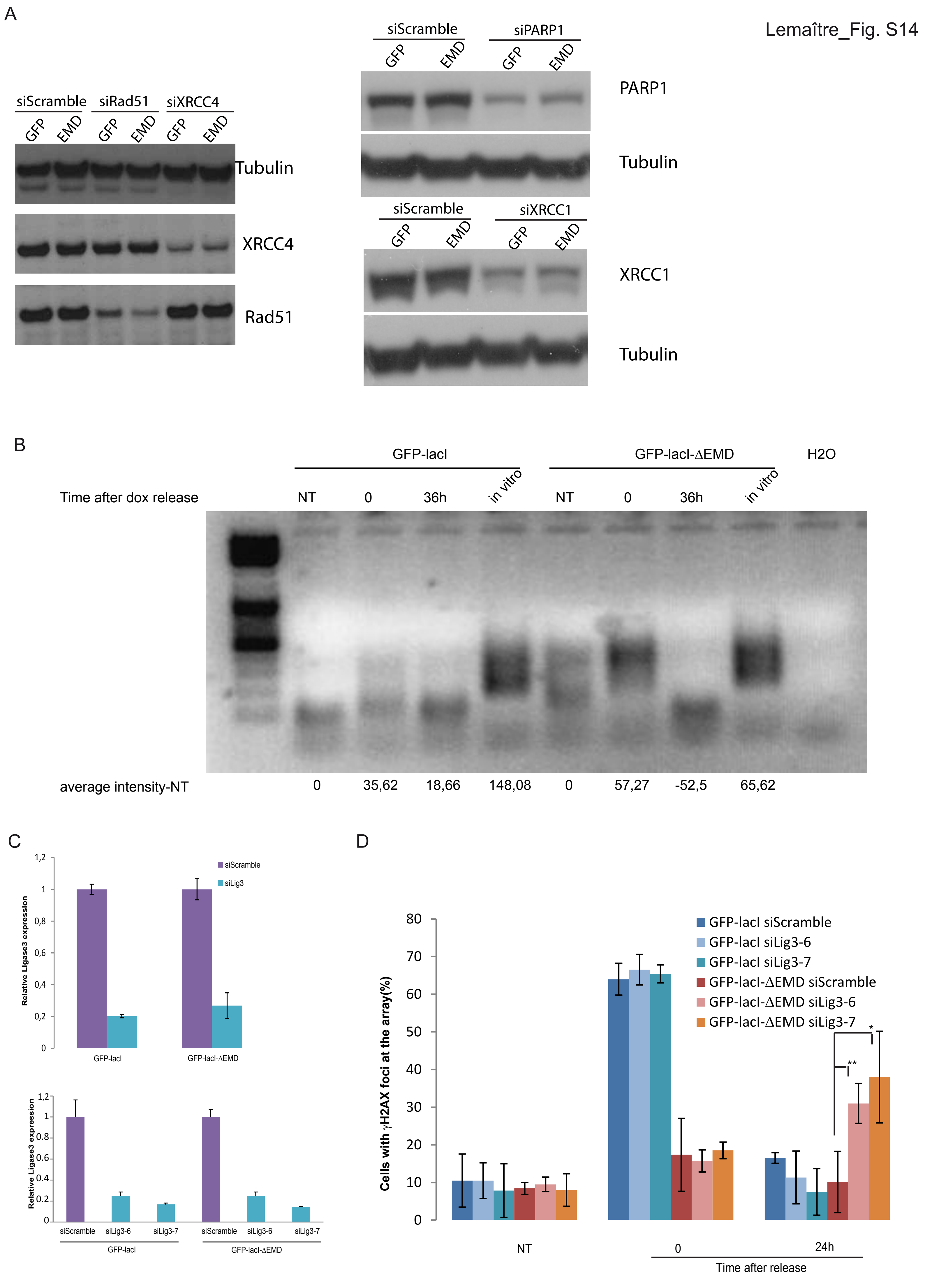

Supplement: Supplemental Material [file supp_gad.248369.114_SuppMaterialREV.pdf]
